# Characterization and modeling of partial-thickness cutaneous injury from debris-simulating kinetic projectiles

**DOI:** 10.1038/s44172-022-00031-6

**Published:** 2022-10-29

**Authors:** Christopher A. Berkey, Omar Elsafty, Montanna M. Riggs, Reinhold H. Dauskardt

**Affiliations:** 1 Department of Materials Science and Engineering, Stanford University, Stanford, CA USA; 2 Department of Mechanical Engineering, Stanford University, Stanford, CA USA

**Keywords:** Mechanical engineering, Tissues

## Abstract

Partial-thickness cutaneous injuries distributed over exposed body locations, such as the face and extremities, pose a significant risk of infection, function loss, and extensive scarring. These injuries commonly result from impact of kinetic debris from industrial accidents or blast weaponry such as improvised explosive devices. However, the quantitative connections between partial-thickness injuries and debris attributes (kinetic energy, shape, orientation, etc.) remain unknown, with little means to predict damage processes or design protection. Here we quantitatively characterize damage in near-live human skin after impact by debris-simulating kinetic projectiles at differing impact angles and energies. Impact events are monitored using high-speed and quantitative imaging to visualize skin injuries. These findings are utilized to develop a highly predictive, dynamic computational skin-injury model. Results provide quantitative insights revealing how the dermal-epidermal junction controls more severe wound processes. Findings can illuminate expected wound severity and morbidity risks to inform clinical treatment, and assess effectiveness of emerging personal protective equipment.

## Abstract (web summary)

Berkey and colleagues quantitatively characterized partial-thickness cutaneous injuries after impact from projectiles simulating ballistic fragments. A corresponding damage model was developed to simulate and predict the cutaneous damage from impact, which could guide protective equipment design and clinical treatment.

## Introduction

Distributed partial-thickness cutaneous injury has serious health consequences, including proliferative scarring and abnormal tissue regeneration with profound adverse functional and cosmetic outcomes. Partial-thickness skin wounds commonly result from accelerated debris clouds associated with the increasing threat of blast weaponry such as mortar shelling and improvised explosive devices^1^. Civil and industrial accidents with explosives or compressed gas can also be causes^2–6^.

The skin has a complex multilayered tissue structure exhibiting a range of mechanical behavior and damage modes. In the case of macroscale mechanical injuries such as predator attack, the tear resistance of full-thickness skin is known to surpass that of other biological tissues due to the unique strength and stretchability of dermal elastin and collagen fiber networks^7–10^. The outer ~200 µm thick epidermis, including the topmost ~15 µm thick stratum corneum, provides mechanical resistance to minor injury while serving as a barrier against microbial pathogens, oxidant stress (ultraviolet light), and chemical compounds^7,11,12^. Subcutaneous fatty tissue dissipates pressure and impacts energy while providing flexibility^13^.

In partial-thickness impact wounds, projectiles may partially penetrate through and damage some skin layers but are prevented from fully penetrating (or perforating) into deeper soft tissue^14^. Partial-thickness wounds may be caused after impact by projectiles traveling more parallel (low-angle impact) or more normal (high-angle impact) to the skin surface.

As regards partial-thickness cutaneous injury from debris impact, the damage processes at work are not well understood. One skin component expected to resist local shear forces in low-angle impacts is the basement membrane known as the dermal-epidermal junction (DEJ). The DEJ is a complex network of proteins and hemidesmosome linkages that provides adhesion and mechanical integrity between the dermis and epidermis^15,16^. There are few, if any, studies with insights into the role of the DEJ in dynamic wound processes.

Skin injuries resulting from dynamic impact, particularly those involving extremities, often present severe bleeding and possible infection risks with potential cascading complications implicated in substantial morbidity and follow-up care^17,18^. Partial-thickness wounds to the face require specialized medical treatment due to high cosmetic and functional importance as well as increased risk to subdermal structures^19,20^. Typical wound management can be costly and requires intensive wound debridement protocols to remove colonized necrotic tissue and foreign bodies. This enhances the formation of healthy granulation tissue and accelerates normal wound healing^18,21,22^.

However, although partial-thickness cutaneous damage presents a significant threat to long-term health, there remains a largely unknown quantitative connection between the type, shape, size, and other parameters of skin wounds and projectile characteristics. These parameters include projectile energy, shape, friction properties, impact angle, and orientation, among others. This gap in knowledge leaves little means to predict and model damage processes, categorize injury severity, or develop standardized treatment procedures accounting for all observed wound morphologies.

During debris impact, large amounts of deformation and energy transfer occur, which may cause tissue crush, abrasion, laceration, puncture, and even partial-thickness avulsion wounds. These wound categories have different levels of severity and treatment protocols depending on size, shape, and body location^4,23,24^. For example, partial-thickness cutaneous injuries located near joints or regions of dynamic mobility have increased significantly since high skin tension challenges wound closure and enhance scarring^25,26^. Wound shape and depth control the degree of scar-forming wound contraction in partial-thickness injuries and whether primary or secondary intention healing should be pursued^27,28^.

Many previous studies have investigated the ballistic impact on soft tissue, including skin, with a strong focus on perforating bullet impacts. Research indicated key parameters controlling injury severity are bullet velocity, shape, mass, and behavior upon entering the tissue (deformation, fragmentation, etc.)^29,30^. The projectile causes a permanent wound cavity, and energy dissipation within the body during impact causes a larger, temporary wound cavity that may also significantly damage surrounding tissue^29–31^. In some cases, equations or linear regressions are developed to correlate projectile velocity to penetration depth or to estimate a perforation velocity threshold^32–35^. During impact, skin provides enhanced perforation resistance to underlying muscle^32^, but is also crushed, lacerated, and harmed by the temporary wound cavity^36,37^. Research evaluating blast debris and fragmenting munition impact also tends to focus on tissue perforation and resulting infection or musculoskeletal risks^21,38–40^. However, there exist surprisingly few studies considering the full biomechanical complexity of human skin in relation to fragment impacts causing non-perforating, partial-thickness cutaneous injury.

A known connection between injury source, related impact parameters, and resulting partial-thickness skin wounds also enables the quantitative development and assessment of personal protective equipment (PPE). By incorporating experimentally observed connections between projectile impact parameters and wound formation, computational models may be developed that simulate an impact event with high fidelity to predict and optimize PPE effectiveness^37,41^. Previously reported ballistic models with lower fidelity concerning the biomechanical complexity of human skin have nonetheless aided PPE design by providing insights such as the human vulnerability and bullet effectiveness at different body locations^42,43^, the threat of behind armor blunt trauma^44^, and the depth of idealized soft tissue penetration by certain ballistic projectiles^45^. However, high-fidelity models of the body, including the skin and constructed with experimental data, represent the ideal platform to test PPE designs^14,41^.

**Fig. 1:**
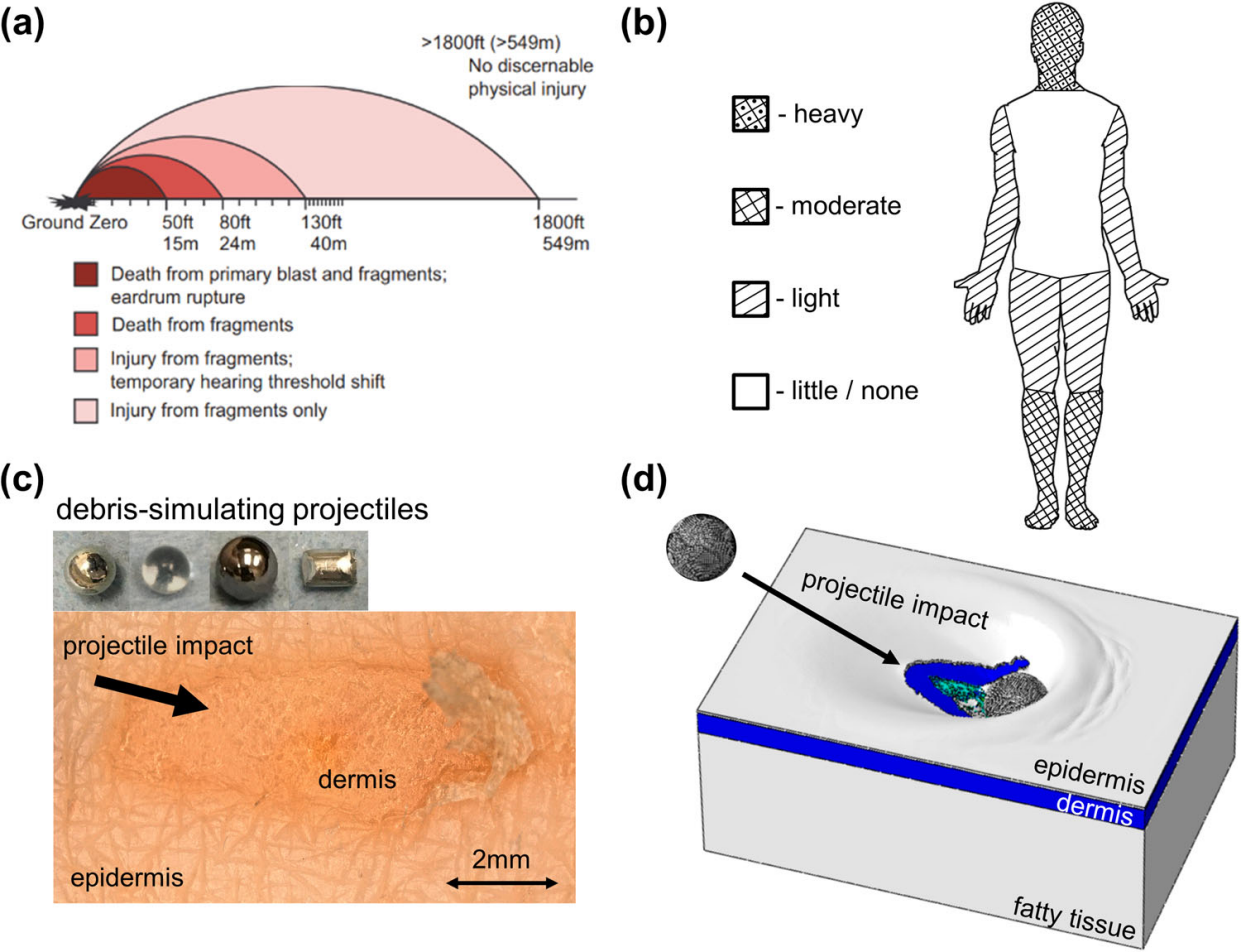
Distributed cutaneous injury overview. **a** Injury outcomes as a function of open space distance from detonation point of a 155 mm (220 lb, ~100 kg) mortar shell^52^. **b** Schematic showing areas prone to partial-thickness skin wounds considering protection from common PPE. **c** Microscope image of impact injury in full-thickness human skin tissue including images of projectiles used to simulate ballistic fragment impact. **d** Computational FE skin impact-injury model includes three main skin layers showing tissue damage and deformation.

The most commonly observed wound source (and the leading cause of injury and death during recent military conflicts) is fragmenting blast weaponry^46–49^. This includes the increased threat of improvised explosive devices, which often produce numerous nonlethal casualties with distributed partial-thickness injury patterns^50,51^. The high volume of partial-thickness wounds occurs because distributed fragment impact is reported as the dominant injury over the largest range from the detonation point (Fig. 1a)^52^. The blast launches numerous projectiles with highly irregular shapes and sizes that travel at high initial velocities in many directions before decelerating^52,53^. Some of these projectiles or accompanying debris can be biologically active, causing increased contamination of distributed partial-thickness skin wounds^54,55^.

As PPE technology has progressed, body armor vests have proven effective at minimizing wounds to the torso and abdomen^56,57^. However, cutaneous wounds to the unprotected and highly significant areas of the head, face, neck, joints, and other mobile extremities remain a critical threat with limited lightweight and flexible PPE solutions. The result is wound density distributions for both perforating and partial-thickness skin injuries similar to that shown schematically in Fig. 1b^51^. Studies of ballistic injury epidemiology do not typically present data with reference to fragment-induced non-perforating wounds. However, reports of external, simple, or minor skin and soft tissue wounds caused by fragments in recent conflicts^57–59^ and attacks^48^ have indicated between 2 and 35% of the total studied casualties present similar injuries to the head, face, neck, or extremities. Often, partial-thickness cutaneous wounds to highly mobile body regions are treated with standard wound closure but typically occur along with more severe trauma, which complicates treatment and outcomes^57,58^. Thin and flexible PPE strategies that are wearable in these highly mobile body regions should be developed with a quantitative assessment of their ability to reduce cutaneous wounds.

In the present work, we quantitatively characterize partial-thickness cutaneous wound size, depth, and shape along with the related damage modes in human skin sections after impact with debris-simulating kinetic projectiles (Fig. 1c). To perform dynamic analysis of the projectile impact, we develop and validate a three-dimensional finite element (FE) computational damage model of full-thickness skin tissue capable of simulating and predicting partial-thickness cutaneous injuries (Fig. 1d). The quantitative insights and predictive capability displayed here have implications for understanding partial-thickness cutaneous damage from projectile and fragment impact to guide clinical treatment and design effective PPE strategies.

## Results

### Spherical projectile cutaneous injury

**Fig. 2:**
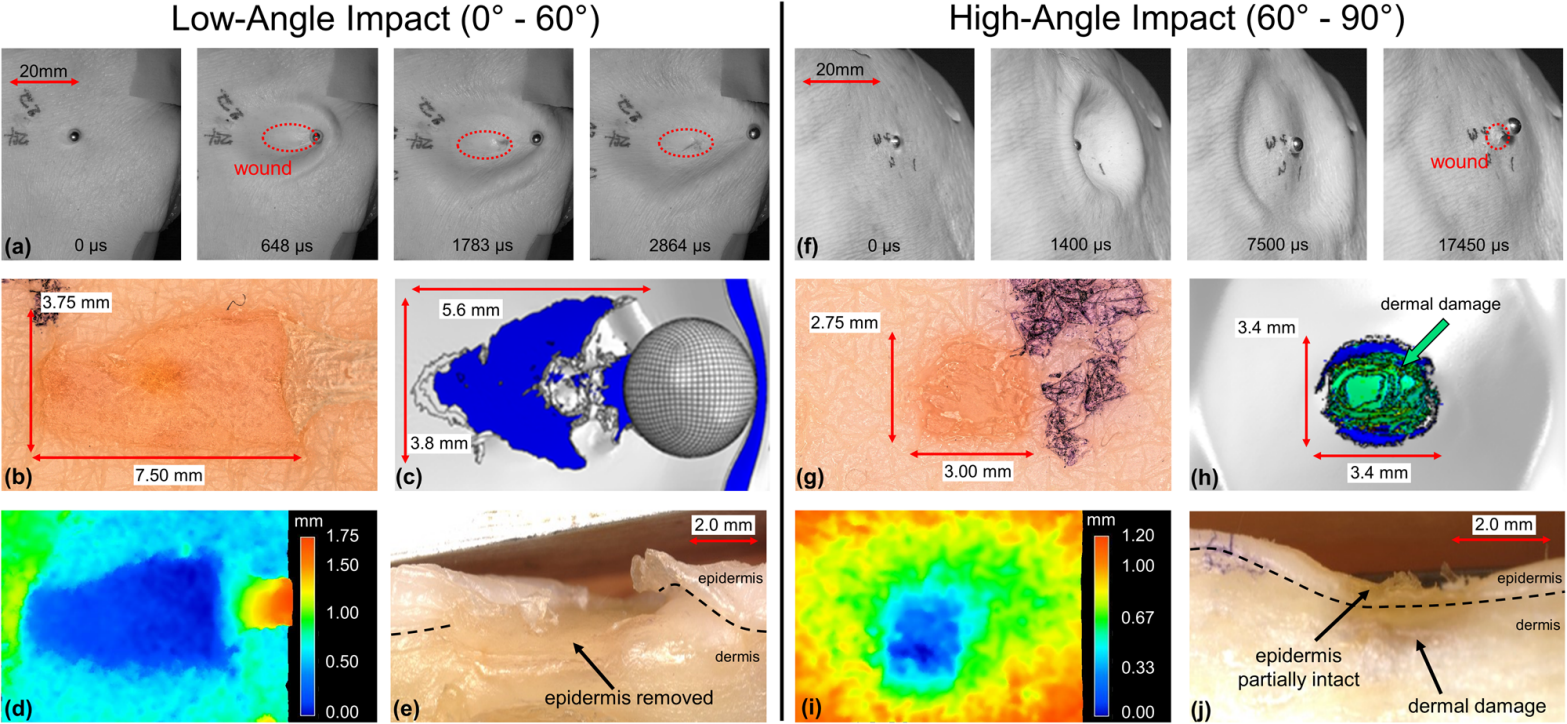
Imaging spherical projectile impact. **a** Selected frames from high-speed video footage of 4.4 mm diameter steel sphere impacting skin tissue at a low angle. **b** Optical microscope image of injury after low-angle impact with 4.4 mm steel sphere. **c** Computed injury from FE impact-injury model after low-angle impact. Optical microscope **d** depth profile and **e** cross-section image of low-angle injury. **f** Selected frames from high-speed video footage of 4.4 mm steel sphere impacting skin at a high angle. **g** Optical microscope image of injury after high-angle impact with 4.4 mm steel sphere. **h** Computed injury from FE impact-injury model after high-angle impact. Optical microscope images of **i** depth profile and **j** cross-section after high-angle injury.

A sequence of images taken from the high-speed visualization of cutaneous injury during a low-angle spherical debris-simulating projectile impact is shown in Fig. 2a. The projectile velocity was 70 ms^−1^ and the projectile kinetic energy per cross-section unit area, called the kinetic energy density (KED), was 5.6 Jcm^−2^. This footage indicates how spherical projectiles initiate delamination of the epidermis along their direction of travel, starting at the point of impact. Additionally, these projectiles cause considerable tissue deformation as they roll along the skin surface.

Digital microscopy of a low-angle impact injury confirms epidermal abrasion with minor dermal damage and reveals a roughly semi-elliptical wound shape with a long axis along the direction of projectile travel (Fig. 2b). The injury width at its largest is 3.75 mm, slightly less than the 4.4 mm projectile diameter, and the total injury area is 23.1 mm^2^. The removed epidermal flap is evident at the right edge of the injury.

Computational simulation shows similar injury characteristics, including epidermal delamination, minor dermal damage at a depth of 0.24 mm, and semi-elliptical wound shape (Fig. 2c). The computational impact sequence shows initial sliding of the sphere causing epidermal delamination, and subsequent rolling of the projectile halts the tearing process and allows the projectile to exit the wound area. Three-dimensional profiling of the injury indicates a depth of about 0.51 mm that does not significantly vary over the injury area (Fig. 2d). A low-angle impact-injury cross-section further verifies the removal of the epidermis, some underlying dermal damage, and consistent injury depth (Fig. 2e).

Injury during a high-angle spherical projectile impact is shown in Fig. 2f. The projectile velocity was 91 ms^−1^ and the KED was 9.3 Jcm^−2^. The projectile creates a large temporary crater on the skin surface before rebounding and disrupting a localized area of the epidermis. The circular wound is 7.4 mm^2^, with roughly equivalent width and length of 2.75 and 3.00 mm, respectively (Fig. 2g). Small lacerations in the epidermis are observed, without the delamination seen for low-angle impacts.

Computational results are similar to the experiment, and also reveal significant permanent dermal damage to a depth of 0.75 mm (Fig. 2h). Depth profiling establishes a crater-like wound, ~0.64 mm deep (Fig. 2i). Injury cross-section exhibits disrupted, partially intact epidermis along with dermal crush damage below the impact site (Fig. 2j).

### Cylindrical projectile cutaneous injury

**Fig. 3:**
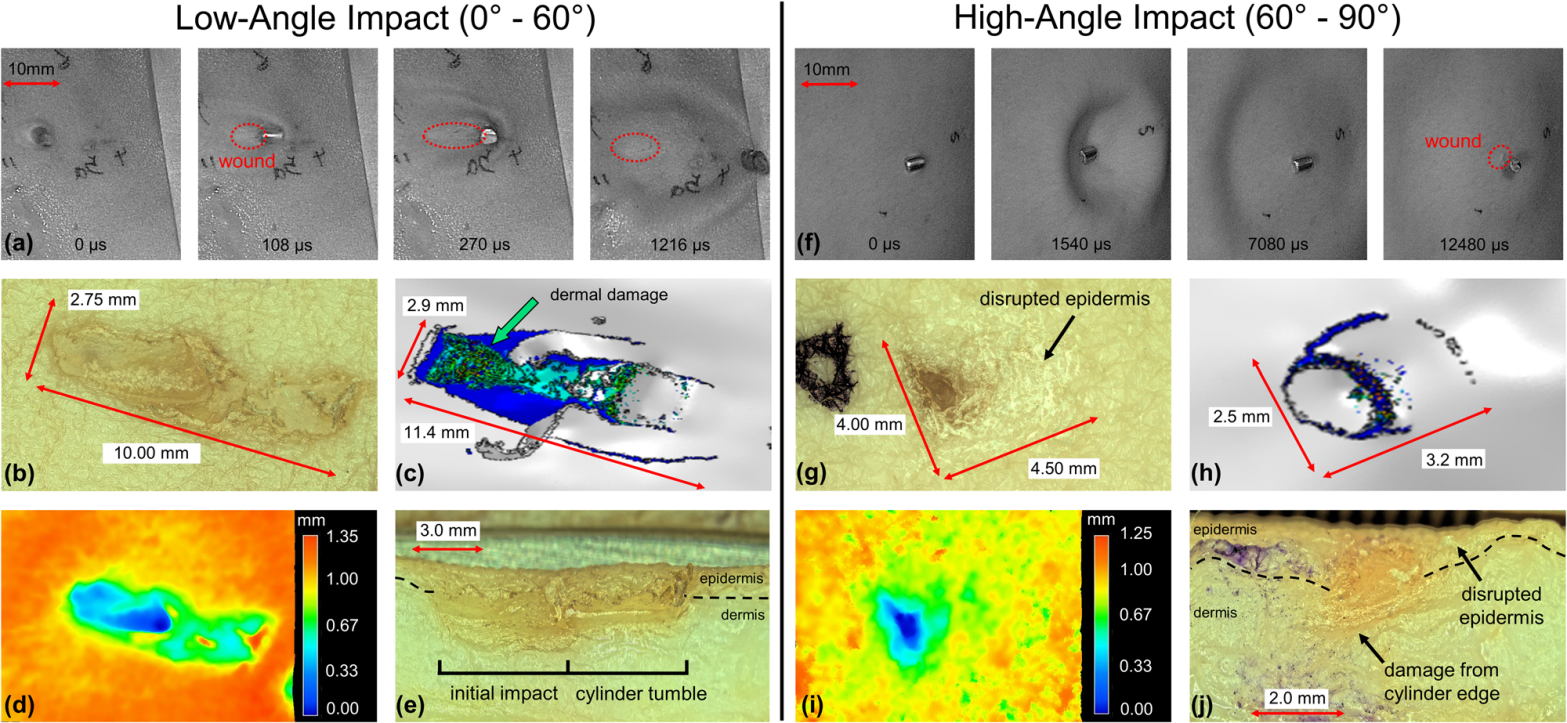
Imaging cylindrical projectile impact. **a** Selected frames from high-speed video footage of 3.2 mm diameter Zn cylinder impacting skin tissue at a low angle. **b** Optical microscope image of injury after low-angle impact with 3.2 mm diameter Zn cylinder. **c** Computed injury from FE impact-injury model after low-angle impact. Optical microscope **d** depth profile and **e** cross-section image of low-angle injury. **f** Selected frames from high-speed video footage of 3.2 mm diameter Zn cylinder impacting skin tissue at a high angle. **g** Optical microscope image of injury after high-angle impact with 3.2 mm diameter Zn cylinder. **h** Computed injury from FE impact-injury model after high-angle impact. Optical microscope images of **i** depth profile and **j** cross-section after high-angle injury.

Low-angle impact with debris-simulating cylinders is shown in Fig. 3a. The projectile velocity was 114 ms^−1^ and the KED was 17 Jcm^−2^. The projectile edge initiates an injury that is extended as the projectile tumbles end over end. Tumbling leads to a 24.9 mm^2^ fusiform injury with a 2.75 mm width (similar to the 3.2 mm cylinder diameter), and a large length of 10 mm along the direction of the tumble (Fig. 3b).

Computation shows a comparable fusiform injury, with more significant epidermal and dermal damage at the point of impact to a depth of 1.1 mm, and reduced damage along the direction of projectile tumble (Fig. 3c). Depth profiling and cross-sectioning demonstrate how cylinder edge impact initially causes a deep 0.95 mm injury into the dermis, before tumbling to produce a shallower 0.43 mm injury extension that is mostly epidermal damage (Fig. 3d, e).

The high-angle cylindrical projectile impact is shown in Fig. 3f. The projectile velocity was 84 ms^−1^, and the KED was 9.2 Jcm^−2^. The cylinder left edge contacts the skin first and disengages last, after the remainder of the cylinder rebounds. The resulting 15.3 mm^2^ injury is mostly epidermal disruption that is roughly circular with width 4 mm and length 4.5 mm (Fig. 3g). However, there is a distinct injury region of deep dermal damage where the left edge of the cylinder is impacted.

FE modeling similarly presents a localized 0.76 mm deep injury region with light damage broadly surrounding it (Fig. 3h). From the model, minimal rolling and sliding effects were detected at this high-angled impact. Depth profiling and injury cross-sectioning uncover a deep 0.73 mm indentation caused by the cylinder left edge and little to no injury depth for the region of the disrupted epidermis (Fig. 3i, j).

### Effects of impact angle and energy on cutaneous injury

**Fig. 4:**
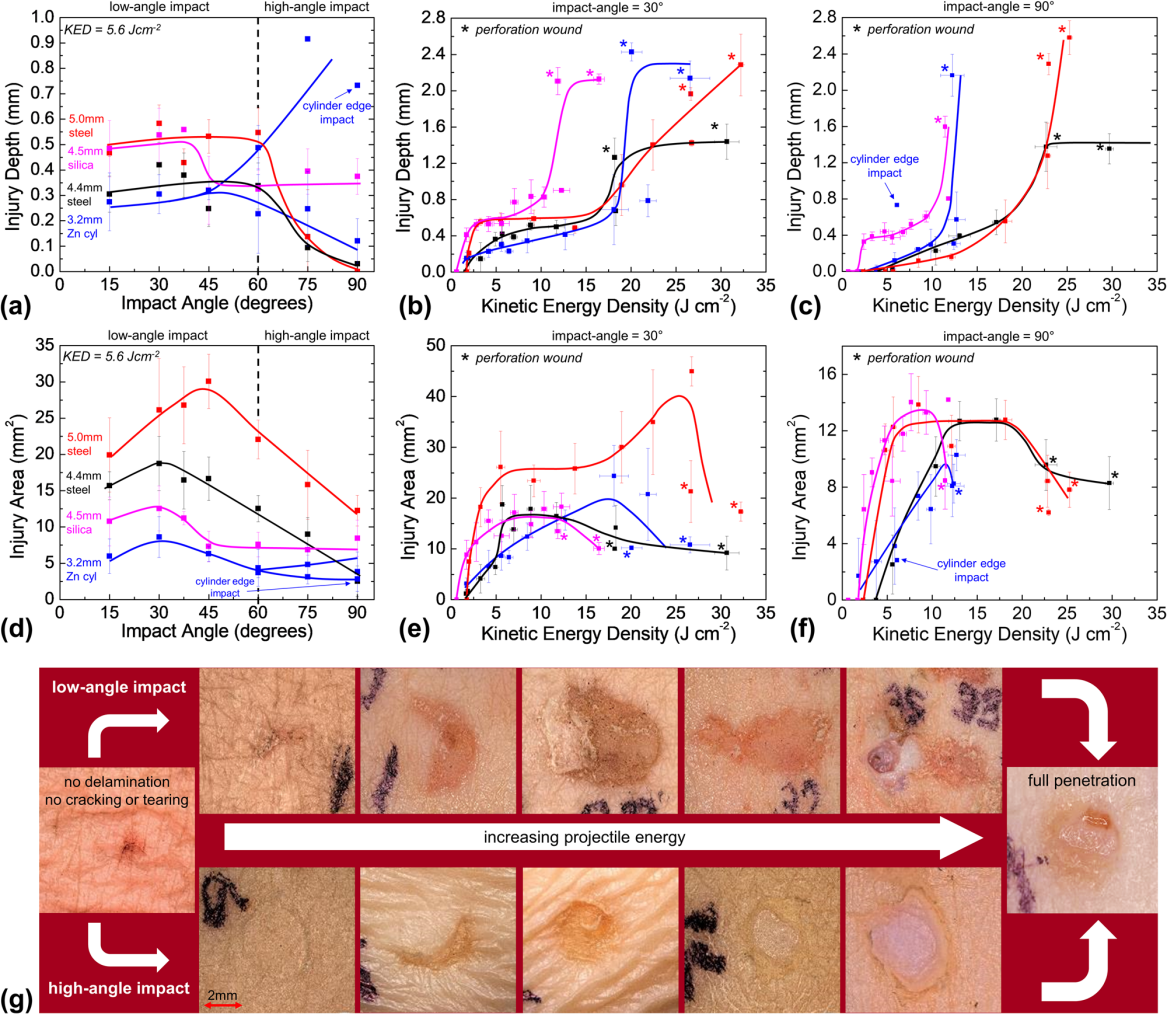
Injury severity dependence on impact energy, angle, and projectile. **a** Injury depth versus projectile impact angle at a KED of 5.6 Jcm^−2^. Injury depth plotted as a function of projectile KED for impact angle of **b** 30° and **c** 90°. **d** Injury area versus projectile impact angle at a KED of 5.6 Jcm^−2^. Injury area plotted as a function of projectile KED for impact angle of **e** 30° and **f** 90°. **g** Collection of typical injury morphologies seen in low and high-angle impacts as a function of increasing projectile KED. The image size is ~8 mm by 8 mm. Graphed data represent nearly 480 unique wounds, with an average *N* value of 4.8 for each data point. Error bars represent standard deviations of average values.

Measurements of injury depth as a function of impact angle for multiple projectile types at a constant KED of 5.6Jcm^−2^ are shown in Fig. 4a. The KED of 5.6 Jcm^−2^ was selected since it was the minimum KED at which non-zero depth and area damage was observed for every projectile. For spherical projectiles, injury depth is maximized for low-angle impacts. Silica spheres cause an elevated injury depth at high angles as well. Injury depth is strongly dependent on cylindrical Zn projectile orientation for high angles, as edge impacts dramatically increase depth compared to face or side impacts. At low angles of 15°, 30°, and 45°, edge impacts observed via high-speed imaging were found to cause injuries of shape and size similar to side and face impacts. Thus, injury characteristics for all orientations were averaged together at these angles.

Depth versus KED for 30° impacts is shown in Fig. 4b. There is a rapid increase in depth at low energies for all projectiles. In the case of cylinders and 4.4 mm diameter steel spheres, depth continued to increase with KED until perforation around 18 Jcm^−2^. By contrast, the 5.0 mm diameter steel spheres showed a plateau in injury depth after the initial increase and penetrate around 27 Jcm^−2^. Depth increased most quickly for silica spheres, with perforation at only 12 Jcm^−2^.

Injury depth versus KED at an impact angle of 90° is shown in Fig. 4c. There is a linear relationship between KED and depth for steel and Zn projectiles until near-perforation energies are reached, and injury depth increases rapidly (23 Jcm^−2^ for both steel sphere sizes and 12.5 Jcm^−2^ for Zn cylinders). A cylinder impact at 5.6 Jcm^−2^ which led to a dramatic depth increase, is labeled as an edge impact, as theorized from computational results. Several cylinder edge impacts were observed experimentally via high-speed imaging, always causing more penetration (or sometimes perforation) compared to other observed impact orientations at similar projectile velocities. Silica sphere injuries immediately increase to a depth of 0.35 mm at the onset of damage, then increase linearly in depth until a sharp increase before perforation at 11.5 Jcm^−2^.

Injury area versus impact angle at constant KED of 5.6 Jcm^−2^ is shown in Fig. 4d. For all projectile types, wound area reaches a maximum at an impact angle between 30° and 45° and tends to decrease at 15°. Larger projectiles produce greater wound areas, especially in low-angle impacts, with the notable exception of silica spheres. The effect of Zn cylinder orientation shows little effect on the injury area.

Injury area versus KED for 30° impacts is shown in Fig. 4e. Larger injury areas are seen compared to 90° impacts. The 5.0 mm steel sphere causes the largest wound area, reaching 45 mm^2^ before perforation. Silica and 4.4 mm steel spheres produce maximum injury areas of 16–18 mm^2^, though as before, the silica projectile reaches its maximum at lower KED of ~4 Jcm^−2^. Injury areas from cylinders increase slower but reach the second largest maximum of ~22 mm^2^ before perforation.

Injury area versus KED for 90° impacts is shown in Fig. 4f. Injury area increases with KED to a maximum before decreasing at the onset of projectile embedding and perforation. Spherical projectiles reach plateaus between 12 and 14 mm^2^, while cylinder injuries quickly peak at 10 mm^2^ before decreasing. The silica and 5.0 mm steel spheres reach maximum area damage at a relatively low KED of ~6 Jcm^−2^, whereas cylinders and 4.4 mm steel spheres reach maximum values at ~12 Jcm^−2^.

A collection of representative images exhibits the observed progression in injury severity with increasing impact energy for low and high-angle impacts (Fig. 4g). The projectiles included steel spheres and Zn cylinders at low (30°) and high (75° and 90°) impact angles with KEDs ranging from 1.4 to 29.0 Jcm^−2^. Low-angle impacts tend to abrade the epidermis at low KED until eventually embedding into and penetrating the dermis. At intermediate KED, epidermal delamination is more extensive, with less of the epidermal flap remaining over the wound bed. By contrast, high-angle impacts cause underlying dermal crush damage and epidermal disruption without delamination at low to intermediate KED. As projectile KED increases further, the epidermis is fully removed with eventual dermal embedding and perforation.

### Computational analysis of projectile orientation and DEJ failure strength

**Fig. 5:**
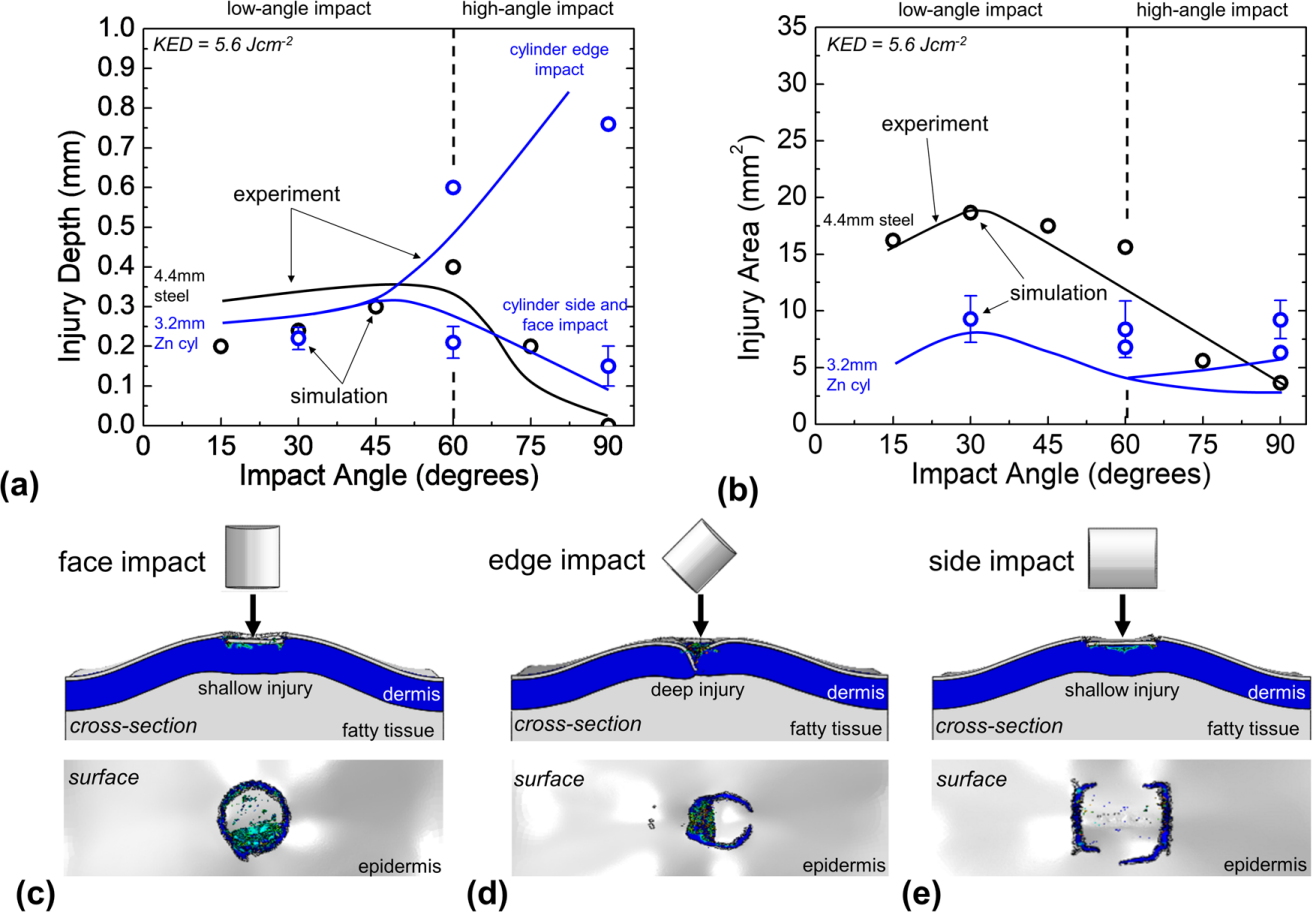
Computation validation and projectile orientation analysis. **a** Simulated wound depth plotted as a function of incident projectile impact angle. **b** Simulated wound area plotted as a function of incident projectile impact angle. Computational results showing cross-section and surface views of injuries immediately following cylinder **c** face impact, **d** edge impact, and **e** side impact at an incident angle of 90°. Simulated injury area and depth values for cylinder face and side impact were averaged together at 60° and 90°, and all orientation values were averaged together at 15° since predicted wounds were similar. Error bars represent the standard deviation in average simulated injury depth and area.

Computational predictions of injury depth and area, made using the three-dimensional FE simulation of cutaneous damage, are shown in Fig. 5a, b. Results from computation strongly agree with all trends in injury size observed experimentally. Statistical comparison of the experimental and computational results provide R^2^ values of 0.71 and 0.93 for the sphere and cylinder injury depth results, respectively, and values of 0.96 and 0.73 for the sphere and cylinder injury area results, respectively.

This agreement includes the divergence of measured injury depths after cylindrical Zn projectile impact. Three separate cylinder orientations are modeled, including face, edge, and side impact, since the nonspherical shape of the projectile is expected to have a strong effect. Simulations of cylinder impact at 15° reveal that cylinder orientation has no significant effect on low-angle wound size or shape, just as was observed experimentally. Thus, predicted injury characteristics for all orientations were averaged together at 15°. At angles of 60° and 90°, simulated face and side impact injuries remained similar and so were averaged together.

Fig. 5c–e shows simulation results of injuries after impact with cylinders in these orientations. Computation indicates that injury depth is strongly dependent on cylindrical Zn projectile orientation for high angles, as edge impacts dramatically increase depth compared to face or side impacts. Low-angle injuries appear insensitive to orientation, as also observed in the FE simulations.

**Fig. 6:**
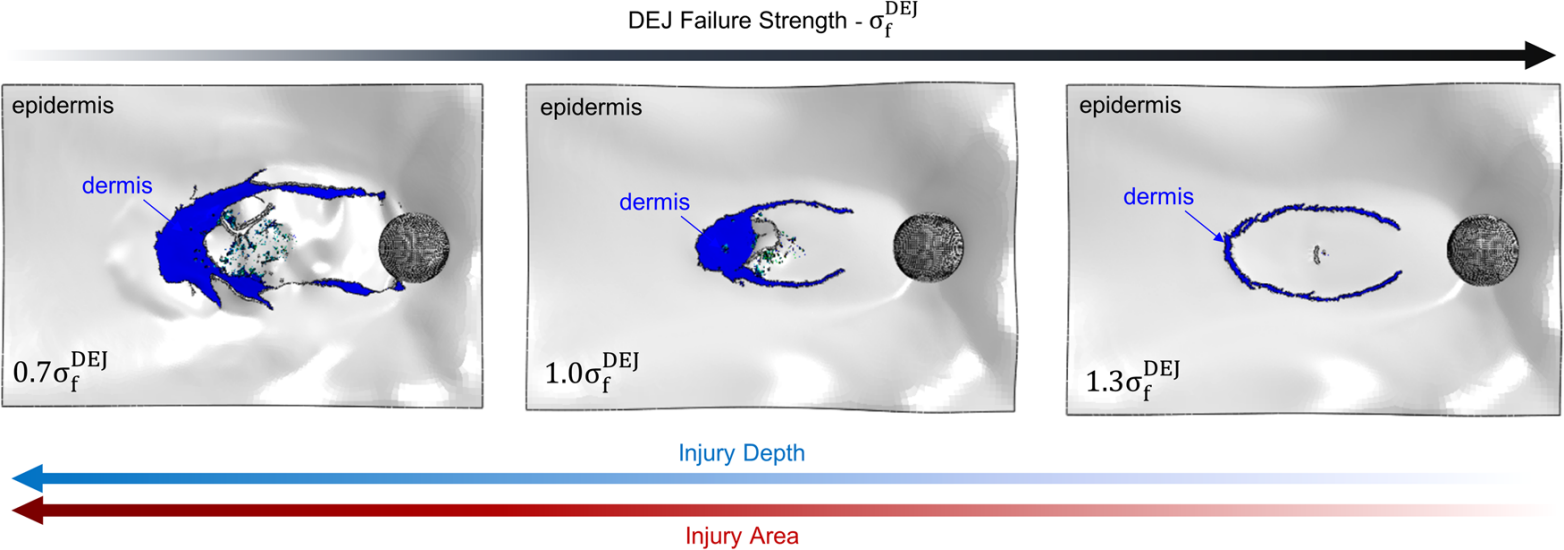
Computation assessment of DEJ role resisting low-angle impact. Computed wounds after spherical projectile impact at 30° and 5.6 Jcm^−2^ for a DEJ failure strength of 0.7σ_f_, 1.0σ_f_, and 1.3σ_f_. All computational model parameters except σ_f_ are held constant between simulations.

Three low-angle impact simulations were performed for a normalized DEJ failure strength (σ_f_) of 0.7σ_f_, 1.0σ_f_, and 1.3σ_f_ (Fig. 6). All computational model parameters except σ_f_ are held constant between simulations. The results illustrate the differences in wound severity when the strength of the DEJ is decreased or increased by 30%. Findings indicate that an increase in the failure strength of the DEJ significantly decreases wound area and depth by inhibiting the partial-avulsion damage mode.

## Discussion

The present study reveals that partial-thickness cutaneous injury from kinetic debris impact involves two key damage mechanisms: abrasion or partial-thickness avulsion controlled by the epidermis and DEJ, and tissue crush controlled by the dermis. Often accompanying both these critical mechanisms are lacerations to initiate injury and partial to full punctures once energy reaches threshold values.

Imaging during spherical steel projectile impact suggests epidermal abrasion dominates in low-angle impacts due to strong shear forces (Fig. 2a–e). However, the epidermal thickness of the abdomen (~0.2 mm) is not enough to account for the injury depths of 0.3–0.5 mm observed at lower KED^60,61^ (Fig. 4a, b). These injury depths indicate deeper dermal tissue, such as the papillary dermis layer is removed along with the epidermis, indicating partial-thickness avulsions occur. This increased depth is attributable to strong adhesion from the high surface area, interdigitated DEJ pulling some of the dermis along as the skin surface is stripped^8,15,62^.

Considering that low-angle impacts also cause large injury areas over all energies, these partial-avulsions present a substantial threat of contamination and bleeding to the exposed dermal tissue^18,22,63,64^. Larger projectiles pose a greater risk, as the 5.0 mm diameter sphere caused markedly enhanced injury areas with increased depths. The DEJ is necessary to avoid skin blistering and maintain skin integrity against low-velocity mechanical insults^16,65^. However, during the avulsion or peeling process caused by the low-angle dynamic impact, the DEJ distributes damage over larger areas and into the papillary dermis.

Tissue crush and epidermal cracking dominate in high-angle spherical steel impacts, where the low amount of projectile velocity parallel to the skin surface cannot cause the shear forces necessary to initiate partial-avulsion. Interestingly, the epidermal lacerations observed after high-angle impact may correspond to similar skin damage described as the abrasion ring or dilation mark in the case of perforating gunshot wounds^66^. In this case, epidermal damage occurs due to the substantial amount of elastic tissue stretch that arises during temporary impact crater formation (Fig. 2f). The temporary wound crater or cavity is a known impact energy dissipation mechanism of the skin and soft tissue that may also cause significant internal damage, especially during perforating impacts^29–31^. The elastic and viscoelastic properties of skin are known to underlie temporary crater formation, and also explain why permanent damage from high-angle impacts is of smaller diameter than the projectile itself in observed partial-thickness and perforating cutaneous damage (Fig. 4f)^30,66^.

For spherical steel projectiles, high-angle injuries show significantly reduced wound depths compared to low-angle impacts. This likely indicates the epidermis and tough dermal collagen (known to resist tearing^9^) are more resistant to crush and tearing from normal forces than the DEJ (known to resist shear^65^) is resistant to shear and partial-avulsion. Consequently, the epidermis and dermis remain relatively intact under high-angle impact until a rapid onset of perforation at large KED when crush and tearing damage modes are activated (Fig. 4a, c).

The results indicate that in the case of non-perforating cutaneous wounds, there is a significant contrast in wound severity between partial-avulsions from low-angle impacts and tissue crush from high-angle impacts. Clinicians should be aware that most injuries presenting as surface abrasions are more similar to partial-avulsion injuries with a significant amount of dermal damage that likely needs primary intention healing, such as tissue adhesives. Testing and assessment of PPE strategies must account for the enhanced partial-thickness wounding present in low-angle impacts to reduce the risk of significant injuries.

Silica sphere injuries are strongly influenced by a lower skin-silica friction coefficient of 0.22–0.28 compared to 0.46 and higher for skin-steel^67,68^. The effects of reduced friction include increased wound depth from low and especially high-angle impacts, reduced wound area in low-angle impacts and reduced thresholds for both damage onset and perforation for all impacts. However, similarities between the injury area of silica and 4.4 mm steel spheres in Fig. 4e may indicate the effect of reduced projectile friction on low-angle impacts could be secondary to projectile size or more dependent on individual tissue characteristics. It appears that reduced friction decreases the interaction between the projectile and skin surface, thus concentrating impact energy and activating dermal tearing damage modes at lower KED. Low friction reduces the amount of applied shear force and diminishes the likelihood of partial-avulsion, but at the cost of enhancing the likelihood of dangerous tissue penetration.

These findings on strong material friction effects provide useful insights to protect the skin. PPE design should minimize significant skin injuries by accounting for a reduced KED threshold for damage when guarding against low-friction materials. Thin PPE development for high-mobility body regions should also consider how different strategies contact the skin and distribute impact energy, and whether partial-avulsion or penetration damage processes are amplified.

An analysis of United Kingdom soldiers wounded by explosive fragments from 2008 to 2011 showed that while spherical blast debris is common, cylindrical shapes are the most often observed^53^. We found the edges of cylindrical projectiles typically caused mixed damage of tissue crush, tearing, and partial-avulsion after low-angle impacts. Deeper dermal tearing is computationally predicted and experimentally observed where cylinder edges impact and lacerate the skin during the projectile tumble. This mixed damage mode leads to thinner injuries with localized areas of deep dermal damage. The lack of extended partial-avulsion and fusiform shape of these injuries make them more likely to heal by secondary intention^27,69^, in contrast with the more significant wounds caused by complete shearing of the DEJ.

High-angle cylinder impacts produce tissue crush injuries that are strongly sensitive to cylinder orientation (edge, side, face) upon impact. Edge-first impacts produce dramatically enhanced wound depths and penetrate the skin at lower KED owing to the concentration of force. Due to the non-ideal nature blast debris clouds, projectiles significantly pitch, yaw, and rotate during travel such that all impact angles, orientations, and associated cutaneous injury may occur^70,71^. As such, emerging PPE designs must account for the increased penetration threat of nonspherical blast debris.

To summarize key experimental findings, we observe at non-perforating projectile velocities that low-angle impacts are usually more aggressive due to the shearing of the DEJ. This partial-avulsion wound mechanism causes not only an increased area of damage but also injury depths reaching the dermis. However, we note low friction and force concentration from projectile edges greatly enhance observed injury depth and perforation risk, especially in high-angle impacts.

One limitation of these experiments is the vast range of additional parameters that may influence wound morphology and severity. For example, the dynamic friction coefficient of skin can be affected by multiple factors such as age, hydration, body location, sex at birth, and applied loading such that a complete ballistic analysis of all these parameters is prohibitively resourced intensive^68,72^. We expect computational capabilities to meet this need in future work, finding that our computational FE impact-injury model was distinctly effective in computing injury mechanisms at different impact angles (partial-avulsion, dermal tearing, etc.) and highly quantitative when predicting injury size. Instead of a computational model, other reports have developed equations to enable the calculation of injury characteristics given projectile parameters^32–35^. However, these equations are usually empirically based on specific datasets and can be challenging to apply to other impact conditions and skin types^32^. The computational model presented in this work includes input parameters that are able to describe a wide range of skin and constituent layer types along with projectile parameters.

This high-fidelity FE model enables the study of those impacts challenging to experimentally evaluate, such as those with different body locations/types, variations in skin hydration or environmental humidity, irregular shapes like stellate octahedra, and controlled distributions of projectiles in a debris cloud. For example, the combined thickness of epidermis and dermis varies with body location such that skin is roughly 0.8–2.0 mm thick around the head, face, and neck^73^, roughly 1.0–3.0 mm thick on the forearm^74^, and reported abdominal tissue thicknesses widely vary between 1.9 and 5.7 mm^75,76^. Thinner skin is expected to present less ballistic resistance, though experimental ballistic assessment of so many different skin thicknesses with tissue from sensitive body locations like the face is prohibitive. The computational capability demonstrated in this work overcomes this challenge since the model, first refined with presently reported experimental results, is then easily tuned to evaluate other body locations.

The effect of cylinder orientation on injury depth is thoroughly understood using computational analysis, due to the difficulty of adjusting projectile orientation at impact experimentally. The model also showed that shear force from sliding friction is the dominant source of epidermal tearing, while rolling friction allows the projectile to exit the wound area with minimal further damage to the skin.

Additionally, the significance of the DEJ was illustrated through computational means by adjusting its failure strength to observe the large changes in injury area and depth. It is critical to note that even while the tear resistance of the epidermis and dermis remains constant, a reduction in DEJ strength dramatically reduces the resistance of the skin to low-angle shearing impacts and the related partial-avulsion damage mode. While shearing damage has been observed under normal conditions in patients afflicted with certain DEJ diseases such as epidermolysis bullosa^16^, the current study now reveals that under dynamic impact conditions, even healthy skin is susceptible to similar damage.

Thus, the DEJ plays a key role in determining whether a partial-thickness impact wound will occur via a less damaging tissue crush mechanism or a more severe partial-avulsion mode. Future work should include continued computational analysis of impact parameters, including variations in the biological and biomechanical properties of skin. These studies would further develop an understanding of how the biophysical structure of the skin aids its function as a complex multilayered protective barrier against projectile impact.

## Conclusions

In conclusion, we elucidated the connection between debris-simulating projectile size, shape, orientation, friction coefficient, impact angle, and KED on partial-thickness skin-injury formation. We found that partial-avulsions dominate in low-angle shear impacts, while dermal tearing mechanisms control high-angle impacts. The DEJ was revealed as a critical skin component that resists the severe partial-avulsion injury mechanism, although damage is extended deeper when the DEJ is sheared from the skin. These experimental findings were complemented with an accurate FE impact-injury model of full-thickness cutaneous tissue. The computational capability determined the same trends found experimentally, demonstrating excellent predictive competency that can be used to further assess the effects of multi-projectile debris cloud impacts, different debris materials, and biological parameters such as age, skin hydration level, sex at birth, and body location. These experimental and computational insights advance the current understanding of partial-thickness skin wounds to support the development and assessment of treatment protocols and thin, flexible PPE for high-mobility body regions.

## Methods

### Tissue preparation

Full-thickness samples of near-live cadaverous abdominal human skin were obtained from nine donors and procured through the National Disease Research Interchange. The National Disease Research Interchange is a 501(c)(3) not-for-profit, National Institutes of Health funded organization that provides project-driven human biospecimen service to academic and corporate scientists. Experiments were conducted on both nonfrozen tissues with low postmortem interval preserved using Dulbecco’s Modified Eagle Medium and tissue stored at −80 °C until needed. Postmortem interval before nonfrozen tissue recovery was between 1 and 5 h. After recovery, samples were immediately placed into a chilled mixture of Dulbecco’s Modified Eagle Medium preservative and antibiotics and then shipped. Shipment time ranged from 24 to 36 h before arrival and immediate testing. Before experiments, the frozen tissue was transferred to a −20 °C freezer. Immediately prior to testing, this tissue was thawed above a water bath at 45 °C for 1 h. No significant differences in experimental results were observed for frozen and nonfrozen tissue samples. In both cases, subcutaneous fat was partially trimmed in areas of excess thickness to level the skin surface, and the tissue was mounted to a ballistic pork gelatin block (BallisticsProGel; BCI) using T-pins. Tension approximating natural skin tension was applied to the skin during mounting with T-pins to remove large tissue undulations and wrinkles and ensure a relatively flat test surface. T-pins were spaced around the tissue edge at an interval of 2–3 cm to prevent large motion of the tissue boundary. Impacts near the skin edge were evaluated and discarded if obvious edge effects were observed, such as the directionality of the wound normal to the edge.

### Kinetic impact system

A kinetic impact system was designed and constructed using components acquired from Airforce Airguns (Fort Worth, Texas). The system accumulates compressed He gas at a chosen pressure and rapidly releases gas to accelerate projectiles loaded into an 18” long barrel. Barrel diameters of 0.177”–0.25” are compatible to enable the use of different size projectiles. An optical chronograph (G2 Precision Chronograph; Caldwell) measures projectile velocity. A mounting stage with translational and angular adjustment allows for precise control over projectile impact position and angle. Debris-simulating projectiles include steel spheres with a diameter of 4.4 mm and a mass of 0.34 g, steel spheres with a diameter of 5.0 mm and a mass of 0.51 g, silica spheres with a diameter of 4.5 mm and a mass of 0.12 g, and Zn cylinders with a diameter 3.2 mm, average length 4.25 mm, and average mass of 0.21 g. Projectile masses are comparable to reported masses of the most common blast debris and fragments^53^. A high-speed camera (Phantom v2512; Vision Research) records the process of projectile impact. KED is calculated by dividing the projectile kinetic energy by the minimum projectile cross-sectional area. For cylindrical projectiles, a true cross-section is not easily defined, given the tendency of all cylinder orientations in low-angle impacts to tumble while making skin contact. In this case, the cylinder face is always used to calculate KED such that the penetration capacity of the projectile is never underestimated for purposes of PPE design.

### Optical microscopy

A digital optical microscope (VHX-7000; Keyence) was used to measure cutaneous damage. The injury area was calculated after defining the injury perimeter to enclose obvious epidermal disruption or color change, and injury depth was determined using three-dimensional profiling. In the case of perforation, injury depth represents the combined thickness of the epidermis and dermis.

### Computational FE impact-injury model

The FE model was developed using ABAQUS EXPLICIT 2019 to accurately simulate and predict partial-thickness skin damage. To account for the mechanical effect of each skin layer, the skin was modeled as a multilayered composite structure composed of the SC, living epidermis, dermis, and subcutaneous fat. Layer thicknesses were calibrated to the near-live cadaverous abdominal human skin tissue used in experiments. The width of the skin model was set to 20 mm. The skin model length was set to 20 mm for high-angle impacts (60°–90°) and 30 mm for low-angle impacts (0°–60°) to account for increased surface interaction occurring in low-angle impacts. The Ogden Hyperelastic constitutive model was used to simulate the material behavior of each layer, and viscoelastic behavior was attributed to the dermis and subcutaneous fat layers using Prony series to account for energy absorption and dissipation during impact. Inverse FE analysis was used to optimize for the damage behavior of the epidermal and dermal layers, as well as the DEJ. A dynamic friction coefficient of 0.46 was applied between the projectile and the skin surface.

The incompressible isotropic Ogden hyperelastic model was used to model the living epidermis and hypodermis with a strain energy density, *W*, written in the form:

1 $$W={\sum }_{i=1}^{N}\frac{2{\mu }_{i}}{{{\alpha }_{i}}^{2}}\left({\overline{{\lambda }_{1}}}^{{\alpha }_{1}}+{\overline{{\lambda }_{2}}}^{{\alpha }_{2}}+{\overline{{\lambda }_{3}}}^{{\alpha }_{3}}-3\right)$$

where *λ*_*1*_, *λ*_*2*_, and *λ*_*3*_ are the principal stretches, *N* is equal to 1, and coefficients *μ*_*i*_ and *α*_*i*_ are temperature-dependent material parameters. Literature values shown in Table 1 were used for the material coefficients of the epidermis and hypodermis^77^.

**Table 1:** Ogden hyperelastic model coefficients.

Skin layer	μ (MPa)	α
Epidermis	2.92	6.35
Hypodermis	0.0104	13.57

The dermis was modeled by the anisotropic Gasser-Ogden-Holzapfel model written in the form:

2 $$W=C(\overline{{I}_{1}}-3)+\frac{{k}_{1}}{2{k}_{2}}{\sum }_{i=4,6}[exp\{{k}_{2}{[\kappa \overline{{I}_{1}}+(1-3\kappa )\overline{{I}_{i}}-1]}^{2}\}-1]$$

where *C*, *k*_1_, and *k*_2_ are temperature-dependent material parameters. $\overline{{I}_{1}}$ is the first deviatoric strain invariant associated with pressure, and $\overline{{I}_{4}}$ and $\overline{{I}_{6}}$ are the invariants relating to the stretches in the fiber directions for two families of collagen fibers. The level of dispersion in the fiber direction is described by *κ*, which is valued between 0 and 1/3. Perfect fiber alignment is depicted by *κ* equal to 0, and a fully random distribution of collagen fibers is described by *κ* equal to 1/3. Material coefficients were obtained from literature data^78^. In this work, *C* was set to 0.01 MPa, *k*_1_ was set to 2.2 MPa, *k*_2_ was set to 0.86, and *κ* was set to 0.27.

Viscoelastic behavior was incorporated in the dermis and hypodermis using the incompressible form of the Prony time series expansion of the dimensionless relaxation modulus, *g*_*R*_:

3 $${g}_{R}(t)=1-{\sum }_{i=1}^{N}{\bar{g}}_{i}^{p}(1-{e}^{-\frac{t}{{{\tau }_{i}}^{G}}})$$

where ${\overline{g}}_{i}^{p}$ and ${{\tau }_{i}}^{G}$ are the modulus ratio and relaxation time for the *i*th term of the Prony series expansion, and whose values (shown in Table 2) were obtained from the literature^79–82^. The outermost stratum corneum layer was modeled using a linear elastic mechanical response with a stiffness modulus of 100 MPa^83,84^.

**Table 2:** Prony time series expansion coefficients.

Skin layer	*g*_1_	*τ*_1_	*g*_2_	*τ*_2_	*g*_3_	*τ*_3_
Dermis	0.09	1.44	0.15	8.63	0.17	52
Hypodermis	0.09	0.294	0.215	9.557	—	—

Displacement boundary conditions were implemented along the base of the hypodermis in the z-plane and perpendicularly along the side surfaces in the x-direction and y-direction. Enhanced hourglass control and 8-node linear hex elements with reduced integration (C3D8R) were used to mesh the model, and element size was reduced until mesh convergence was achieved. Mesh density was largest at the area of impact, and the smallest element size was 0.02 mm for the SC, 0.05 mm for epidermis and dermis, and 0.1 mm for the hypodermis. A dynamic explicit analysis was performed for a 1.5 ms duration, calculation of the minimum time step was automated to ensure numerical convergence, and dynamic mass scaling was implemented to reduce the computational cost, ensuring that energy conservation was maintained in the process.

### Statistics and reproducibility

To ensure statistical results, ballistic data includes nearly 480 impacts with unique resulting cutaneous wounds. Projectile velocity, injury area, and injury depth were measured for each wound. There are 100 unique data points presented, thus the average *N* value is 4.8 for each data point. The *N* value ranged from 1 to 2 for uncommon perforation wounds to over 10 when analyzing the effect of impact angle and the transition between cutaneous damage mechanisms, though was most often between 3 and 7. Error bars represent standard deviations of average values.

### Reporting summary

Further information on research design is available in the Nature Research Reporting Summary linked to this article.

### Supplementary information

Dauskardt_PR File

Reporting Summary

## Data Availability

The datasets generated and/or analyzed during the current study are available from the corresponding author on reasonable request.
