# Supplementary material for: Characterization and modeling of partial-thickness cutaneous injury from debris-simulating kinetic projectiles
Source: Commun Eng. 2022 Oct 29;1:33. doi: 10.1038/s44172-022-00031-6 (PMC10956024; doi:10.1038/s44172-022-00031-6)
Supplement: Supplementary file 1 — Dauskardt_PR File [file 44172_2022_31_MOESM1_ESM.pdf]

nature portfolio

Peer Review File

Characterization and modeling of partial-thickness cutaneous injury from debris-simulating kinetic projectiles

Open Access This file is licensed under a Creative Commons Attribution 4.0 International License, which permits use, sharing, adaptation, distribution and reproduction in any medium or format, as long as you give appropriate credit to the original author(s) and the source, provide a link to the Creative Commons license, and indicate if changes were made. In the cases where the authors are anonymous, such as is the case for the reports of anonymous peer reviewers, author attribution should be to 'Anonymous Referee' followed by a clear attribution to the source work. The images or other third party material in this file are included in the article's Creative Commons license, unless indicated otherwise in a credit line to the material. If material is not included in the article's Creative Commons license and your intended use is not permitted by statutory regulation or exceeds the permitted use, you will need to obtain permission directly from the copyright holder. To view a copy of this license, visit http://creativecommons.org/licenses/by/4.0/.

Reviewers' comments:

Reviewer #1 (Remarks to the Author):

Comments for the authors of COMMSENG-22-0067:

In brief, the paper deals with the mechanics cutaneous injuries from different shape debris, spherical and cylinders. A tentative to connect the quantitative relationship between partial-thickness injuries and debris attributes (velocity, shape and orientation) is performed, based on both experimental tests and finite element modeling.
Rather sound results were reported in this research. The content is sufficiently original and useful in the clinical treatment and emerging personal protective equipment development. However, the paper needs a MAJOR revision to become completely suitable for publication. The following comments should be addressed:

1) Abstract: It is said in the last sentence that "Findings can inform clinical treatment, guide development of non-lethal weapons…."
I suggest the authors explain a bit more this statement. How can these results help in the clinical treatment? Is the development of non-lethal weapons a question related with good practices, ethical issues?

MAIN TEXT:
2) The paper format and section order should be changed:
First, a "INTRODUCTION" section, for the first part of the paper. It may be from line 34 to 115.
Then, "MATERIALS AND METHODS" , "COMPUTACIONAL FE IMPACT-INJURY" MODEL", and the two final sections: RESULTS and DISCUSSION AND CONCLUSIONS.

3) Some of the parameters and abbreviations are not defined at any point in the text. A key note with all the nomenclature used defined (DEJ, IED, NLWs, KE, …) before the introduction should be included.

4) From line 81, it is said that "A known connection between injury source, related impact parameters, …. of PPE",-
New references must be included to reinforce this statement.

5) Line 103: "The ability to connect projectile parameters to injury size, related wound severity, and relevant skin damage mechanisms is necessary to quantifiably assess the effectiveness of emerging PPE solutions and the significance of skin damage caused by 106 developing NLWs".

New references and more explanation about models that connect the projectile parameters with injuries characteristics.

6) Line 109: "with by". Correct it.

RESULTS:
7) Spherical Projectile Cutaneous Injury: Fig. 2 shows high speed images of cutaneous injury during an impact. The velocity of the projectile is not mentioned for those images. The authors must write it in the text.

8) Text in lines 136 to 140:
" Injury during a high-angle spherical projectile impact is shown in Fig. 2f. The projectile creates a large temporary crater on the skin surface before rebounding and disrupting a localized area of the epidermis. The circular wound is 7.4mm2, with roughly equivalent width and length of 2.75mm and 3.00mm, respectively (Fig. 2g). Small lacerations in the epidermis are observed, without the delamination seen for low-angle impacts. "

Is the high angle impact less aggressive than the low angle impact? This results is about the area, but what about the depth of the injury? Are the velocities of the projectiles equal for both images? Can the damage be considered as volume of damage instead of area damage? Will this difference change the results a lot? Please, explain.

9) Cylindrical Projectile Cutaneous Injury
The dimensions of the cylinder must be described in the text. Are they 3.2mm diameter and 10 mm length?

10) Graphs showing Injury depth and injury area vs. impact angle are presented in the paper. But, even there is a lot of work done, this is only the values obtained in the tests, Could the authors suggest an explanation based on the skin morphology, layers, fibers, …for these results? Can the authors obtain an equation to calculate the injury characteristic depending on impact parameters (velocity, geometry, …)?

11) Computational Analysis of Projectile Orientation and DEJ Failure Strength
Why did the authors choose 0.7σf, 1.0σf, and 1.3σf as values for the DEJ? Is it based in some previous studies?

12) The material models used for skin is Odgen hyperelastic constitutive model and viscoelastic behaviour. The authors must write the constitutive equations of these models and stated the values used for each layer and each model.

Reviewer #2 (Remarks to the Author):

General Comments:
The study is investigating the partial-thickness skin injury from impact by projectiles at different impact angles and energies. Experiments were performed on human skin pinned to a block of ballistic gelatine. A dynamic computational model was developed and validated against the experimental results. From the study, the authors identified the key wounding mechanisms of the partial-thickness skin injury and the role of DEJ in the process and the difference in injury outcomes between low and high angle non-perforating impacts. The computational model showed good agreement with the experimental observations and can be useful in further studies.
The biggest strength of the study is the wealth of the experimental data with quantitative injury assessment. The limitation of the study is the choice of the abdominal skin which can be different to skin of the face and extremities that have high injury frequency. The manuscript should also provide more details on the experimental set-up.

Specific Comments:
INTRODUCTION
The introduction needs to provide quantitative details from literature studies reporting injury frequency, distribution and consequence of non-perforating skin injury which is the focus of this research, especially if the author recommends that PPE "should be developed with quantitative assessment of their ability to reduce cutaneous wounds" later on.
There has been a wide range of research studying ballistic impacts on skin. It is recommended to include a brief summary of the existing literature and highlight the areas or gaps that the present research can address.
In addition, for the benefits of the readers, the authors should define the key terms of the manuscript in this early stage, for example: partial-thickness wound, impact angles, low-angle impacts, high-angle impacts, penetration of the skin [although I think that 'perforation' is a more accurate term for the intended use later in this manuscript, or at least 'full penetration' is more appropriate. This paper: https://militaryhealth.bmj.com/content/159/4/265.short can be a good resource.], etc.

Line 36-38: Please indicate the permission and consent for including identifiable personal details in the publication.
RESULTS
What were the impact velocity in the pairs of examples shown for low and high angle impacts (Fig.2A&F, Fig.3A&F)? Are they comparable?
Please provide the scale for Fig.2C,E,H&J and Fig.3C,E,H&J.
Line 141-142: How is the permanent dermal damage produced by FE model compared to experiment, quantitatively, for these examples?
Line 167: "cross-section KE" – how was this calculated? Did the calculation take into account the different impact orientations?
What was the case for 5.6Jcm-2 chosen as the example? Was it representative for most impact energies? Figure 4E shows that at this energy, the data point for silica sphere was lower than 4.4mm steel sphere whereas it is higher or similar at other energies. This questions how representative Figure 4D is.
Did edge impact occur (was it observed) for low impact angles?
Line 174: "until penetration" – it is more accurate to use the term "perforation" here (and in similar occasions later on), or "full penetration" to avoid confusion with the cases where there was penetration in the skin (hence injury depth), but not full penetration.
[There is a confusion among published studies between skin penetration (the full skin thickness is not necessarily passed through) and skin perforation (the full thickness of the skin is passed through, and a hole should be seen at the back) making it hard to use or reproduce existing data. It is good practice to define what the term(s) means in the paper early on for future applications of the study.]
Line 181-182: Please clarify if the edge impacts were verified experimentally using video footage.
Line 202-203: What was the projectile, its impact velocity and angles used in this example? Please provide the scale for these images in Fig.4G.
Figure 5A&B: Were the data point from a single run or average? What do the error bars represent?
Line 212-214: Were the experimental and computational trends statistically compared? In Fig.5B, there is discrepancy between the results from simulation and experiment, please comment on this.
DISCUSSION
Line 233-240: Please comment on the thickness of the skin layers at the face and extremities which are the area with higher injury frequency, and how the results obtained from the abdominal skin in this study can be applied there.
Line 251-252: Please provide references for "…the epidermis and tough dermal collagen are more resistant to crush and tearing from normal forces than the DEJ is resistant to shear and partial avulsion."
Line 266: "increased wound depth from high-angle impacts" – is it not also the case for low-angle impacts? Fig.4A-C shows that 4.5mm silica sphere produced deeper injury than 4.4mm steel sphere in most cases, including low angles.
Line 266-267: "reduced wound area in low-angle impacts" – it seems to be the case only for 5.6Jcm-2 KE (Fig.4D). Please show that it is also the case for other impact energies. At 30-degree (Fig.4E) and 90-degee (Fig.4F) impacts, 4.5mm silica sphere shows higher injury areas than 4.4mm steel sphere for most KE.
MATERIALS AND METHODS
Line 348-349: How long was the post-mortem time for non-frozen samples?
Line 354: Please clarify that it is ballistic gelatine that was used and what type of gelatine, of what concentration?
Line 354: Was the skin sample mounted under tension? How was the natural skin tension accounted for? Please comment on the boundary condition of the skin sample in the experiment.
Line 356-365:
What was the range of projectile impact velocity used in this study? What was the mass of the projectiles? Are they comparable to the study by Breeze et al. mentioned in the Discussion? Please comment on the relevance of the impact conditions used in this study to clinical cases and realistic scenarios.
Please clarify how the yaw was controlled and measured, and how it was taken into account for cross-

section KE calculations.

Reviewer #3 (Remarks to the Author):

This is an extremely well designed, conducted and presented study investigating the nature of skin wounds inflicted by debris-simulated spherical and cylindrical projectiles at low impact velocities. The authors should be commended upon this study which addresses important issues on the type of injury produced by such projectiles, and the predictive character of projectile characteristics, including kinetic energy, shape, and angle of attack (orientation), with respect to the resultant skin damage. Despite being common, both in armed conflicts and in cases of industrial accidents, this type of relatively superficial ballistic trauma has not been studied in such an extensive manner previously, as the relevant literature mostly deals with penetrating fragment injuries from explosive devices. Consequently, the paper would be of interest not only to engineers involved in the study of ballistics but also to healthcare personnel with an interest in ballistic trauma, war surgery and disaster medicine.

Below are my comments on the text of the manuscript.

1. The authors state that "typical wound management involves antibiotics" (rows 62-63). This is not true. Antibiotics are generally given as prophylactic therapy to prevent infection in contaminated wounds, but not routinely in the case of relatively superficial (shallow) wounds, such as those under study. In reference #21 of the manuscript (Singer & Dagum, 2008), under "General Principles of Care", it is stated that "Prophylactic systemic antibiotics should not be used routinely". Also, in reference #22 (Trott, 2012), it is stated on page 3 "Currently, there are no solid, evidenced-based data showing that antibiotics prevent traumatic skin-wound infections", and also on page 225 "For small, uncomplicated, minor, non-bite wounds and lacerations, there is no convincing clinical evidence that systemic antibiotics provide protection against the development of wound infection".
With respect to facial injuries, in "Maxillofacial Trauma and Esthetic Facial Reconstruction", 2nd edition (by Ward Booth, Elsevier; 2012), on page 432 it is stated that "Routine systemic antibiotics are not necessary. The use of antibiotics should be tailored based on host defense status, degree of contamination, mechanism of injury, and likelihood of wound infection.[...] Prophylactic antibiotics are necessary if the wound is expected to be contaminated, such as animal bites or wounds that communicate with the oral cavity".
The use of antibiotics in skin wounds from debris and small projectiles depends on the depth of the wound (relatively superficial skin wounds such as abrasion injuries, although contaminated, they do not predispose to deep infection) and whether attempts to remove foreign bodies from facial wounds (in the case of traumatic tattooing) are undertaken carrying the risk of further contamination of a superficially contaminated wound.
Regarding skin war wounds in general, In "Military Dermatology" (1994, United States Army, https://irp.fas.org/doddir/milmed/milderm.pdf), on page 149, it is stated "The use of antibiotics to prevent clinically apparent infections may be helpful in some instances".

2. The interesting observations regarding high-angle spherical projectile impact that it is associated with creation of a large temporary crater on the skin surface, while the permanent wound is smaller in diameter than the projectile itself (because of the skin elasticity), also associated with small lacerations in the epidermis (apparently from the stretching effect during the impact) are in accordance with similar effects produced at the site of entrance of bullets. Please refer to Thali et al., "A study of the morphology of gunshot entrance wounds, in connection with their dynamic creation, utilizing the ‘‘skin–skull–brain model’’", Forensic Science International (2002) 125:190–194 (https://pubmed.ncbi.nlm.nih.gov/11909662/). So I would recommend that the authors comment on these similarities, which are both consistent with the skin properties and the process of energy transfer by the projectile upon impact.

3. The authors use the term cross-section kinetic energy (KE) measured in joules per square centimeter (kinetic energy per unit area). A more common term for this measure used in the wound ballistics literature, which is an expression of the penetration capacity of the projectile, is the kinetic energy density. Please refer to Kneubuehl's "Wound Ballistics" (Springer, 2011), and also to the chapter "Personal Armour Used by UK Armed Forces and UK Police Forces" by Lewis et al., in "Ballistic Trauma", 4th edition (Breeze et al., Springer, 2017), and also to Caister et al., "The ballistic performance of bone when impacted by fragments", International Journal of Legal Medicine (2020) 134:1387–1393 (https://pubmed.ncbi.nlm.nih.gov/32358725/).

4. The book "Wounds and Lacerations: Emergency Care and Closure" by Alexander Trott, which appears as reference #22, is in its 4th edition.

We thank the reviewers for their comments on our manuscript submitted to *Communications Engineering*.

The reviewer's comments are listed below together with our detailed responses.

Modifications within the manuscript submitted separately are highlighted in red therein.

Reviewer #1

1. "Abstract: It is said in the last sentence that 'Findings can inform clinical treatment, guide development of non-lethal weapons….' I suggest the authors explain a bit more this statement. How can these results help in the clinical treatment? Is the development of non-lethal weapons a question related with good practices, ethical issues?"

   Response*:* We agree this statement in the Abstract should be better clarified. Findings on the nature of partial-thickness skin wounds inflicted by debris-simulating projectiles are useful in the clinical treatment context by informing relevant healthcare personnel about likely injury severity and associated morbidity risks (scarring, loss of function, etc.). We believe the systematic description of damage mechanisms provided in the manuscript will also give healthcare personnel useful quantitative information to further develop wound treatment strategies in preparation for rapid response to ballistic trauma, war surgery, or disaster medicine.

   Regarding non-lethal weapons (NLWs), our intent was to elucidate the risks of non-perforating skin injuries to ensure the potential damage of these weapons was known and avoided during design. A NLW that severely harms the skin and risks morbidity is not acceptable. However, we appreciate the ethical issues raised by the reviewer in discussing NLWs and have removed from the manuscript all statements concerning NLWs. To clarify the usefulness of results to clinical treatment and removed discussion of NLWs, the final sentence of the Abstract now reads as follows:

   At lines 29-32: *"Findings can illuminate expected wound severity and morbidity risks to inform clinical treatment and assess effectiveness of emerging personal protective equipment."*

2. "The paper format and section order should be changed:
   First, a "INTRODUCTION" section, for the first part of the paper. It may be from line 34 to 115. Then, "MATERIALS AND METHODS", "COMPUTATIONAL FE IMPACT-INJURY" MODEL", and the two final sections: RESULTS and DISCUSSION AND CONCLUSIONS."

   Response*:* The currently implemented format and section order is chosen to conform with the Communications Journals' Style and Formatting Guide. The heading "MAIN TEXT" has been changed to "Introduction" and "Materials and Methods" has been changed to "Methods" to better reflect guidelines.

3. "Some of the parameters and abbreviations are not defined at any point in the text. A key note with all the nomenclature used defined (DEJ, IED, NLWs, KE, …) before the introduction should be included."

***Response**:* Per the Communications Journals' Style and Formatting guide, "no list or table of abbreviations/definitions is permitted." To enhance readability, we have removed those abbreviations that were only used very few times (i.e. "UV" ultraviolet; "NDRI" National Disease Research Interchange; "NIH" National Institutes of Health; "3D" three-dimensional; "DMEM" Dulbecco's Modified Eagle Medium). All abbreviations are now defined after first use.

4. "From line 81, it is said that 'A known connection between injury source, related impact parameters, …. of PPE', - New references must be included to reinforce this statement."

   ***Response**:* Additional clarification and references have been added to support the usefulness of an impact-injury model to develop and assess PPE. References included are [Breeze, J., et al. Determining the wounding effects of ballistic projectiles to inform future injury models: A systematic review. J. R. Army Med. Corps (2014)] and [Breeze, J., Fryer, R., Pope, D. & Clasper, J. Injury representation against ballistic threats using three novel numerical models. J. R. Army Med. Corps (2017)]. Clarification has been added to the Introduction:

   At lines 102-5: *"By incorporating experimentally observed connections between projectile impact parameters and wound formation, computational models may be developed that simulate an impact event with high-fidelity to predict and optimize PPE effectiveness[37,41]."*

5. "Line 103: 'The ability to connect projectile parameters to injury size, related wound severity, and relevant skin damage mechanisms is necessary to quantifiably assess the effectiveness of emerging PPE solutions and the significance of skin damage caused by 106 developing NLWs.' - New references and more explanation about models that connect the projectile parameters with injuries characteristics.

   Response: We agree and have included references to other ballistic models that have helped analyze topics such as the vulnerability of different body locations, the risk of behind armor blunt trauma from non-perforating impacts, and soft tissue penetration. However, few, if any, other reported models have attained the same high level of fidelity to skin mechanics and biological structure. The following additional explanation has been added to the Introduction:

   At lines 105-11: *"Previously reported ballistic models with lower fidelity concerning the biomechanical complexity of human skin have nonetheless aided PPE design by providing insights such as the human vulnerability and bullet effectiveness at different body locations[42,43], the threat of behind armor blunt trauma[44], and the depth of idealized soft tissue penetration by certain ballistic projectiles[45]. However, high-fidelity models of the body, including the skin and constructed with experimental data, represent the ideal platform to test PPE designs[14,41]."*

6. "Line 109: 'with by'. Correct it."

   ***Response**:* We appreciate the reviewer catching this error and have removed "by" at line 146.

7. "Spherical Projectile Cutaneous Injury: Fig. 2 shows high speed images of cutaneous injury during an impact. The velocity of the projectile is not mentioned for those images. The authors must write it in the text."

***Response**:* The reviewer is correct that these velocities (and attendant kinetic energy densities) are useful in analysis of the high-speed images. The key purpose of these images was to elucidate and highlight the main damage mechanisms as we describe again in response to Reviewer #2 comment 5. The projectile velocities and energy densities have been included into the Results section just after reference to the high-speed images:

At lines 156-8: *"The projectile velocity was 70ms^{-1} and the projectile kinetic energy per cross-section unit area, called the kinetic energy density (KED), was 5.6Jcm^{-2}."*

At lines 175-6: *"The projectile velocity was 91ms^{-1} and the KED was 9.3Jcm^{-2}."*

8. "Text in lines 136 to 140:

   'Injury during a high-angle spherical projectile impact is shown in Fig. 2f. The projectile creates a large temporary crater on the skin surface before rebounding and disrupting a localized area of the epidermis. The circular wound is 7.4mm2, with roughly equivalent width and length of 2.75mm and 3.00mm, respectively (Fig. 2g). Small lacerations in the epidermis are observed, without the delamination seen for low-angle impacts.'

   Is the high angle impact less aggressive than the low angle impact? This results is about the area, but what about the depth of the injury? Are the velocities of the projectiles equal for both images? Can the damage be considered as volume of damage instead of area damage? Will this difference change the results a lot? Please, explain"

   ***Response**:* In this point, the reviewer raises several questions about how to interpret and compare injury characteristics between low and high-angle impacts. To first answer specific questions, the injury depths of both the low and high-angle impacts shown in Fig. 2 are already written in the Results section as 0.51mm (line 172) and 0.64mm (line 183), respectively. Additionally, the projectile velocities are not equal because images were selected to highlight key damage mechanisms, as noted in response to Comment #7 from Reviewer #1. The cited Results section text from the manuscript was not commenting on which damage mode is more severe, but rather indicating that in high-angle impacts the delamination and avulsion of skin layers is not observed. The Discussion section already addresses the relative aggressiveness of low and high-angle impacts throughout, such as at lines 324-38, 366-8, and 422-4.

   To now answer the broader questions, we considered but ultimately chose not to evaluate wound volume because it would obscure the severity of observed wounds. This is because a perforating impact with a small permanent wound tract may have a small volume of damaged skin but would often be considered a very severe injury due to internal damage risks. Likewise, a large area wound with a shallow depth localized to the epidermis is likely less severe than a moderate area and moderate depth wound harming the dermis where blood vessels are located, although both wounds could have the same volume. At the projectile velocities studied here, we find low-angle impacts are usually more aggressive because they shear the DEJ and show not only increased area damage but also injury depths reaching the dermis. However, it is important to note low friction and stress concentration from cylinders greatly enhance injury depth and perforation risk, especially in high-angle impacts. To highlight this important conclusion, we have added the following comment into the Discussion section:

At lines 376-81: *"To summarize key experimental findings, we observe at non-perforating projectile velocities that low-angle impacts are usually more aggressive due to shearing of the DEJ. This partial-avulsion wound mechanism causes not only an increased area of damage but also injury depths reaching the dermis. However, we note low friction and force concentration from projectile edges greatly enhance observed injury depth and perforation risk, especially in high-angle impacts."*

9. "The dimensions of the cylinder must be described in the text. Are they 3.2mm diameter and 10 mm length?"

   ***Response**:* The dimensions of all projectiles are already listed in the Methods section with the cylinder dimensions included as 3.2mm diameter and 4.25mm length (line 475).

10. "Graphs showing Injury depth and injury area vs. impact angle are presented in the paper. But, even there is a lot of work done, this is only the values obtained in the tests, Could the authors suggest an explanation based on the skin morphology, layers, fibers, …for these results? Can the authors obtain an equation to calculate the injury characteristic depending on impact parameters (velocity, geometry, …)?"

    ***Response**:* In fact, the reviewer's comment on the experimental limitations was our key motivation for using the experimental data to develop and validate the reported more general skin impact-injury model. In the Discussion section, we already describe the usefulness of the skin impact-injury model to evaluate numerous other types of impacts beyond those experimentally tested here (lines 382-9) and we discuss how the model can also capture unique aspects of skin morphology such as the role of the DEJ (lines 414-21). Whereas an equation developed with experimental data (in the literature these are usually empirically based models) would not be easily translatable to other experimental conditions (i.e. skin hydration, body location, multi-projectile impact interactions, etc.), these experiments are readily performed with our computational model. To stress the greater utility of our model over an equation for determining injury characteristics, we have added a brief comment on other ballistic penetration equations and their limitations in the Discussion section:

    At lines 389-95: *"Instead of a computational model, other reports have developed equations to enable calculation of injury characteristics given projectile parameters[32–35]. However, these equations are usually empirically based on specific data sets and can be challenging to apply to other impact conditions and skin types[32]. The computational model presented in this work includes input parameters that are able to describe a wide range of skin and constituent layer types along with projectile parameters."*

11. "Why did the authors choose 0.7σf, 1.0σf, and 1.3σf as values for the DEJ? Is it based in some previous studies?"

    ***Response**:* The DEJ failure strength was computationally observed to be the most significant parameter in predicting the partial-avulsion wound mechanism and injury characteristics of low-angle impacts as noted lines 414-24. We selected values 30% greater and lower than the implemented DEJ failure strength to best showcase visually the importance of this specific morphological feature of skin with regard to ballistic impact and wound severity.

12. “The material models used for skin is Odgen hyperelastic constitutive model and viscoelastic behaviour. The authors must write the constitutive equations of these models and stated the values used for each layer and each model.”

***Response**:* We agree with the reviewer that more information concerning the material models of the skin, especially the constitutive equations, are helpful to the reader for assessing the skin impact-injury model. Further details concerning the computational model have been added into the Methods section:

At lines 504-27: *“The incompressible isotropic Ogden hyperelastic model was used to model the living epidermis and hypodermis with a strain energy density, W, written in the form:*

$$W = \sum_{i=1}^{N} \frac{2\mu_i}{{\alpha_i}^2}(\overline{\lambda_1}^{\alpha_1} + \overline{\lambda_2}^{\alpha_2} + \overline{\lambda_3}^{\alpha_3} - 3) \quad (1)$$

where λ_1, λ_2, and λ_3 are the principal stretches, N is equal to 1, and coefficients μ_i and α_i are temperature-dependent material parameters. Literature values were used for the material coefficients of the epidermis and hypodermis[77].

The dermis was modeled by the anisotropic Gasser-Ogden-Holzapfel model written in the form:

$$W = C(\overline{I_1} - 3) + \frac{k_1}{2k_2}\sum_{i=4,6}[exp\{k_2[\kappa\overline{I_1} + (1-3\kappa)\overline{I_\iota} - 1]^2\} - 1] \quad (2)$$

where C, k_1, and k_2 are temperature-dependent material parameters. $\overline{I_1}$ is the first deviatoric strain invariant associated with pressure, and $\overline{I_4}$ and $\overline{I_6}$ are the invariants relating to the stretches in the fiber directions for two families of collagen fibers. The level of dispersion in the fiber direction is described by κ, which is valued between 0 and 1/3. Perfect fiber alignment is depicted by κ equal to 0 and a fully random distribution of collagen fibers is described by κ equal to 1/3. Material coefficients were obtained from literature data[78].

Viscoelastic behavior was incorporated in the dermis and hypodermis using the incompressible form of the Prony time series expansion of the dimensionless relaxation modulus, g_R:

$$g_R\,(t) = 1 - \sum_{i=1}^{N} \bar{g}_i^p\,(1 - e^{-\frac{t}{{\tau_i}^G}}) \quad (3)$$

where $\bar{g}_i^p$ and ${\tau_i}^G$ are the modulus ratio and relaxation time for the i-th term of the Prony series expansion, and whose values were obtained from the literature[79–82]. The outermost stratum corneum layer was modeled using a linear elastic mechanical response with a stiffness modulus of 100 MPa[83,84].”

Reviewer #2

1. “The introduction needs to provide quantitative details from literature studies reporting injury frequency, distribution and consequence of non-perforating skin injury which is the focus of this research, especially if the author recommends that PPE ‘should be developed with quantitative assessment of their ability to reduce cutaneous wounds’ later on.

   Response: We agree that information concerning non-perforating skin injuries would be helpful, and we have included some references reporting head, face, neck, and extremity injury frequency from conflicts in Mogadishu [Mabry, R. L. *et a.l, J. Trauma* **49**, 515–529 (2000)], in Lebanon and Israel

[Levin, L. *et al., J. Oral Maxillofac. Surg.* **66**, 1630–1633 (2008)], in Operations Enduring and Iraqi Freedom [Lew, T. A., *et al,. J. Oral Maxillofac. Surg.* **68**, 3–7 (2010)] and in the Madrid train bombing attack [Turégano-Fuentes, F. *et al., World J. Surg.* **32**, 1168–1175 (2008)]. As the reviewer may know, categorization and wound details in most studies evaluating injury frequency are often not reported in a standardized manner, making it difficult to find specific references to "partial-thickness" injuries from blast fragments. Thus, we interpreted mentions of "external," "simple," or "minor" skin wounds as similar or equivalent to "partial-thickness" cutaneous wounds.

Regarding distribution, the previously included reference [Breeze, J., *et al., J. R. Army Med. Corps* **161**, 36–41 (2015)] describes both the distribution of perforating fragment injuries and more superficial injuries in which projectiles were removed via "scrubbing." We expect perforating fragments would not be easily removed via scrubbing and so interpret these wounds as partial-thickness. Additionally, partial-thickness and perforating fragment wounds would be expected to have a similar distribution since fragments of both low and high velocity are expected from a blast event. Indeed, in the cited injury frequency references, treatment and outcomes were muddied by the co-occurrence of partial-thickness wounds with perforating and other more severe injuries. We have included this information into the Introduction:

At lines 125-34: *"The result is wound density distributions for both perforating and partial-thickness skin injuries similar to that shown schematically in Fig. 1c[51]. Studies of ballistic injury epidemiology do not typically present data with reference to fragment-induced non-perforating wounds. However, reports of "external," "simple," or "minor" skin and soft tissue wounds caused by fragments in recent conflicts[57–59] and attacks[48] have indicated between 2 – 35% of the total studied casualties present similar injuries to the head, face, neck, or extremities. Often, partial-thickness cutaneous wounds to highly mobile body regions are treated with standard wound closure, but typically occur along with more severe trauma which complicates treatment and outcomes[57,58]."*

2. There has been a wide range of research studying ballistic impacts on skin. It is recommended to include a brief summary of the existing literature and highlight the areas or gaps that the present research can address."

Response: We agree that a summary of previous skin impact research helps highlight the key advancements of the present research, namely, consideration of the full biomechanical complexity of human skin in relation to non-perforating, partial-thickness cutaneous injury from fragment impacts. To this end, we have included the following brief summary into the Introduction along with several pertinent references:

At lines 86-99: *"Many previous studies have investigated ballistic impact on soft tissue including skin, with a strong focus on perforating bullet impacts. Research indicated key parameters controlling injury severity are bullet velocity, shape, mass, and behavior upon entering the tissue (deformation, fragmentation, etc.)[29,30]. The projectile causes a permanent wound cavity, and energy dissipation within the body during impact causes a larger, temporary wound cavity that may also significantly damage surrounding tissue[29–31]. In some cases, equations or linear regressions are developed to correlate projectile velocity to penetration depth or to estimate a perforation velocity threshold[32–35]. During impact, skin provides enhanced perforation resistance to underlying muscle[32], but is also crushed, lacerated, and harmed by the temporary wound cavity[36,37]. Research evaluating blast debris and fragmenting munition impact also tends to focus on tissue perforation and resulting infection or*

musculoskeletal risks[21,38–40]. However, there exist surprisingly few studies considering the full biomechanical complexity of human skin in relation to fragment impacts causing non-perforating, partial-thickness cutaneous injury."

3. "In addition, for the benefits of the readers, the authors should define the key terms of the manuscript in this early stage, for example: partial-thickness wound, impact angles, low-angle impacts, high-angle impacts, penetration of the skin [although I think that 'perforation' is a more accurate term for the intended use later in this manuscript, or at least 'full penetration' is more appropriate. This paper: https://militaryhealth.bmj.com/content/159/4/265.short can be a good resource.], etc."

   ***Response**:* We agree and thank the reviewer for the provided reference that lays out this terminology and highlights the need for physical and numerical ballistic models of skin such as the model described in this report. We further agree that "perforation" is a more accurate term rather than "penetration" for much of the discussion in this manuscript. Thus, in many cases "penetration" was changed to "perforation," and the following brief description of terminology was added in the Introduction:

   At lines 53-6: *"In partial-thickness impact wounds, projectiles may partially penetrate through and damage some skin layers, but are prevented from fully penetrating (or perforating) into deeper soft tissue[14]. Partial-thickness wounds may be caused after impact by projectiles traveling more parallel (low-angle impact) or more normal (high-angle impact) to the skin surface."*

4. "Line 36-38: Please indicate the permission and consent for including identifiable personal details in the publication."

   ***Response**:* We licensed and obtained the image (Fig. 1a) and descriptive caption, including identifiable personal details, from the Associated Press. After inquiry to the Associated Press, they have confirmed no additional permission is required beyond licensing to publish the image and details. However, in the interest of privacy concerns, we have removed the subject's name replacing it with "A 41-year old Ukrainian citizen…" in the Figure 1 caption.

5. "What were the impact velocity in the pairs of examples shown for low and high angle impacts (Fig.2A&F, Fig.3A&F)? Are they comparable?"

   ***Response**:* The reviewer is correct that these velocities (and attendant kinetic energy densities) are useful in analysis of the high-speed images. The key purpose of these images was to elucidate and highlight the main damage mechanisms operating in the skin during low and high-angle impacts. Because the damage mechanisms associated with different projectiles and impact angles activate and progress at different impact energies, images were selected based on their quality and clarity rather than for comparable projectile velocity. The projectile velocities and energy densities have been included into the Results section just after reference to the high-speed images:

   At lines 156-8: *"The projectile velocity was $70ms^{-1}$ and the projectile kinetic energy per cross-section unit area, called the kinetic energy density (KED), was $5.6Jcm^{-2}$."*

   At lines 175-6: *"The projectile velocity was $91ms^{-1}$ and the KED was $9.3Jcm^{-2}$."*

At lines 186-7: *"The projectile velocity was 114ms^{-1} and the KED was 17Jcm^{-2}."*

At lines 197-8: *"The projectile velocity was 84ms^{-1} and the KED was 9.2Jcm^{-2}."*

6. "Please provide the scale for Fig.2C,E,H&J and Fig.3C,E,H&J."

   Response: Scale bars and measurements of injury dimensions have been included in Fig.2C,E,H&J and Fig.3C,E,H&J.

7. "Line 141-142: How is the permanent dermal damage produced by FE model compared to experiment, quantitatively, for these examples?"

   Response*:* Permanent dermal damage predicted by the FE model was included in model outputs of quantitative injury depth in these examples and in Figure 5a. To show that computed injury depth (and permanent dermal damage) was quantitatively comparable to the already reported experimental values, we have now included in the Results section the computed injury depths for the example simulated wounds shown in Fig. 2c (line 168), Fig. 2h (line 182), Fig. 3c (line 192), and Fig. 3h (line 203).

8. "Line 167: 'cross-section KE' – how was this calculated? Did the calculation take into account the different impact orientations?"

   Response*:* The cross-section KE (or kinetic energy density) is calculated by dividing the projectile kinetic energy by the minimum projectile cross-sectional area. In the case of spherical projectiles, this calculation is straightforward, and the result is more significant than projectile velocity or kinetic energy for determining the penetration capacity. For cylindrical projectiles, the minimum cross-section of the cylinder face is always used such that the penetration capacity of the projectile is never underestimated for purposes of PPE design. We believe the use of this cross-section is additionally appropriate given the tendency of all cylinder orientations in low-angle impacts to tumble while making skin contact, such that a "true" cross-section is not easily defined. Clarification of kinetic energy density calculations is now included in Methods:

   At lines 478-83: *"KED is calculated by dividing the projectile kinetic energy by the minimum projectile cross-sectional area. For cylindrical projectiles, a "true" cross-section is not easily defined given the tendency of all cylinder orientations in low-angle impacts to tumble while making skin contact. In this case, the cylinder face is always used to calculate KED such that the penetration capacity of the projectile is never underestimated for purposes of PPE design."*

9. "What was the case for 5.6Jcm-2 chosen as the example? Was it representative for most impact energies? Figure 4E shows that at this energy, the data point for silica sphere was lower than 4.4mm steel sphere whereas it is higher or similar at other energies. This questions how representative Figure 4D is."

   Response*:* A kinetic energy density (KED) of 5.6Jcm-2 was chosen since this was the minimum KED at which depth and area damage occurred for every projectile at every angle. 5.6Jcm-2 is roughly the KED threshold for non-zero depth damage to occur from 90° impacts of 4.4mm and 5.0mm diameter steel spheres (Figure 4c). We selected the minimum KED damage threshold for a scan of angle

effects to highlight partial-thickness damage mechanisms caused by low velocity projectiles. The damage mechanisms observed at this KED were highly representative of all impacts.

In Figure 4e the area damage from silica spheres is also less than 4.4mm steel spheres at 8.9Jcm-2 and 11.7Jcm-2, not only at 5.6Jcm-2. We believe biological diversity between tissue donors used in these separate experiments explains the few mm^2 variations in silica sphere injury areas. The resulting Figure 4e similarities in injury area may indicate the low-friction effect of silica displayed in Figure 4d is secondary to projectile size and does not always dominate in low-angle impacts for every donor. We have clarified the selection of 5.6Jcm-2 in the Results section and commented on Fig. 4e similarities in the Discussion:

At lines 210-2: *"The KED of 5.6Jcm^{-2} was selected since it was the minimum KED at which non-zero depth and area damage was observed for every projectile."*

At lines 343-6: *"However, similarities between injury area of silica and 4.4mm steel spheres in Fig. 4e may indicate the effect of reduced projectile friction on low-angle impacts could be secondary to projectile size or more dependent on individual tissue characteristics."*

10. "Did edge impact occur (was it observed) for low impact angles?"

    ***Response**:* Edge impact of cylinders at low angles was observed along with face and side impacts via high-speed imaging. Interestingly, there were no significant differences observed due to cylinder orientation in low-angle impact wounds, so values were averaged together. We clarify this in the Results section:

    At lines 215-8: *"At low angles of 15°, 30°, and 45°, edge impacts observed via high-speed imaging were found to cause injuries of shape and size similar to side and face impacts. Thus, injury characteristics for all orientations were averaged together at these angles."*

11. "Line 174: "until penetration" – it is more accurate to use the term "perforation" here (and in similar occasions later on), or "full penetration" to avoid confusion with the cases where there was penetration in the skin (hence injury depth), but not full penetration. [There is a confusion among published studies between skin penetration (the full skin thickness is not necessarily passed through) and skin perforation (the full thickness of the skin is passed through, and a hole should be seen at the back) making it hard to use or reproduce existing data. It is good practice to define what the term(s) means in the paper early on for future applications of the study.]"

    ***Response**:* We agree with the reviewer on the use of perforation and have discussed defining terms earlier in response to Comment #3 from Reviewer #2.

12. "Line 181-182: Please clarify if the edge impacts were verified experimentally using video footage."

    ***Response**:* The actual occurrence of cylinder orientation effects is important to stress, and indeed, several cylinder edge impacts were observed experimentally via high-speed imaging that always caused more penetration (and sometimes perforation) compared to other observed impact

orientations at similar projectile velocities. This clarification is added into the Results section where the cylinder edge impact is mentioned:

At lines 230-2: *"Several cylinder edge impacts were observed experimentally via high-speed imaging, always causing more penetration (or sometimes perforation) compared to other observed impact orientations at similar projectile velocities."*

13. "Line 202-203: What was the projectile, its impact velocity and angles used in this example? Please provide the scale for these images in Fig.4G."

    ***Response**:* We have included the following Fig. 4g details in the Results section and figure caption:

    At lines 255-7: ***"****The projectiles included steel spheres and Zn cylinders at low (30°) and high (75° and 90°) impact angles with energies ranging from 1.4 to 29.0 Jcm^{-2}."*

    In the Fig.4g caption: "*The image size is approx. 8mm by 8mm.*"

14. "Figure 5A&B: Were the data point from a single run or average? What do the error bars represent?"

    ***Response**:* One simulation of cylinder impact was performed for each of the three described cylinder orientations (side, edge, and face). We found that at 15° there was no significant difference in simulated wound characteristics, just as was found experimentally, so these results were averaged together. At angles of 60° and 90°, simulated face and side impact injuries remained highly similar and so were averaged together. Error bars represent the standard deviation of the simulated average values. To clarify this data, the following comments were added into the Results section and the Figure 5 caption:

    At lines 274-8: *"Simulations of cylinder impact at 15° reveal that cylinder orientation has no significant effect on low-angle wound size or shape, just as was observed experimentally. Thus, predicted injury characteristics for all orientations were averaged together at 15°. At angles of 60° and 90°, simulated face and side impact injuries remained similar and so were averaged together."*

    In Fig. 5 caption: *"Simulated injury area and depth values for cylinder face and side impact were averaged together at 60° and 90°, and all orientation values were averaged together at 15° since predicted wounds were similar. Error bars represent the standard deviation in average simulated injury depth and area."*

15. "Line 212-214: Were the experimental and computational trends statistically compared? In Fig.5B, there is discrepancy between the results from simulation and experiment, please comment on this."

    ***Response**:* Comparison of the experimental and computational results provide R^2 values of 0.71 and 0.93 for the sphere and cylinder wound depth results, respectively, and values of 0.96 and 0.73 for the sphere and cylinder wound area results, respectively. Overall, this is remarkable agreement between experiment and a computational model that is not a fit but based on reported literature values. Additionally, all injury size trends with angle, KED, and shape observed experimentally were the same when predicted computationally. The slight discrepancies in injury area values between some of the

experimental and computational results are likely due to biological differences between tissue donors or small variations in cylinder orientation at impact. Comments on the R^2 values are now added to the Results section:

At lines 267-70: *"Statistical comparison of the experimental and computational results provide R^2 values of 0.71 and 0.93 for the sphere and cylinder injury depth results, respectively, and values of 0.96 and 0.73 for the sphere and cylinder injury area results, respectively."*

16. "Line 233-240: Please comment on the thickness of the skin layers at the face and extremities which are the area with higher injury frequency, and how the results obtained from the abdominal skin in this study can be applied there."

    ***Response**:* We had previously mentioned the strength of the computational model in assessing other body locations that are difficult to experimentally evaluate due to tissue size or sensitivity constraints (lines 396-9). The key point is the model may be easily applied to assess other body locations by tuning skin layer thickness and mechanical properties based on literature reports. Thus, after refining and validating the model on thicker abdominal skin, it may be applied to locations such as the face and extremities. These comments are added into the Discussion section along with some references to skin thicknesses of the abdomen, face, and forearm:

    At lines 399-408: *"For example, the combined thickness of epidermis and dermis varies with body location such that skin is roughly 0.8 – 2.0mm thick around the head, face and neck[73], roughly 1.0 – 3.0mm thick on the forearm[74], and reported abdominal tissue thicknesses widely vary between 1.9 – 5.7mm[75,76]. Thinner skin is expected to present less ballistic resistance, though experimental ballistic assessment of so many different skin thicknesses with tissue from sensitive body locations like the face is prohibitive. The computational capability demonstrated in this work overcomes this challenge since the model, first refined with presently reported experimental results, is then easily tuned to evaluate other body locations."*

17. "Line 251-252: Please provide references for "…the epidermis and tough dermal collagen are more resistant to crush and tearing from normal forces than the DEJ is resistant to shear and partial avulsion."

    ***Response**:* We have provided references describing the role of dermal collagen in resisting tearing forces and the DEJ in resisting shearing forces. Our intent in this statement was to report the implications of a key finding of spherical steel impacts. Namely, because larger injury depths occur after low-angle impacts at the same KED as high-angle impacts, then the skin must be less resistant to shearing than normal forces. We have clarified this point with minor changes to the cited statement, such that it now reads in the Discussion as:

    At lines 324-9: *"For spherical steel projectiles, high-angle injuries show significantly reduced wound depths compared to low-angle impacts. This likely indicates the epidermis and tough dermal collagen (known to resist tearing[9]) are more resistant to crush and tearing from normal forces than the DEJ (known to resist shear[65]) is resistant to shear and partial avulsion. Consequently, the epidermis and dermis remain relatively intact under high-angle impact until a rapid onset of perforation..."*

18. “Line 266: “increased wound depth from high-angle impacts” – is it not also the case for low-angle impacts? Fig.4A-C shows that 4.5mm silica sphere produced deeper injury than 4.4mm steel sphere in most cases, including low angles?”

    Response*:* We agree the silica produced deeper injury compared to 4.4mm steel sphere at low-angles, though point out the significance of depth increase is much greater in high-angle impacts (Fig. 4c). To acknowledge the friction effects on low-angle impacts we have edited the cited statement in the Discussion:

    At line 341: *“…increased wound depth from low and especially high-angle impacts…”*

19. “Line 266-267: ‘reduced wound area in low-angle impacts’ – it seems to be the case only for 5.6Jcm-2 KE (Fig.4D). Please show that it is also the case for other impact energies. At 30-degree (Fig.4E) and 90-degee (Fig.4F) impacts, 4.5mm silica sphere shows higher injury areas than 4.4mm steel sphere for most KE.”

    Response*:* In Figure 4e the area damage from silica spheres is also less than 4.4mm steel spheres at 8.9Jcm-2 and 11.7Jcm-2, not only at 5.6Jcm-2. We further address this point in response to Comment #9 from Reviewer #2. Additionally, we do not claim nor expect reduced wound area in the 90° impacts (Fig.4f). Low friction enables greater penetration at lower KED such that injury area increases as a larger cross-section of the projectile is impacted into the tissue.

20. “Line 348-349: How long was the post-mortem time for non-frozen samples?”

    Response*:* In experiments on non-frozen samples, post-mortem interval before tissue recovery was between 1 – 5 hours. After recovery, samples were immediately placed into a chilled mixture of Dulbecco’s Modified Eagle Medium preservative and antibiotics and then shipped. Shipment time between tissue recovery and lab testing ranged from 24 – 36 hours. We believe the combination of low PMI and preservative medium make these non-frozen samples highly representative of *in vivo* tissue properties. The additional details concerning post-mortem interval are added into the Methods section:

    At lines 450-4: *“Post-mortem interval before non-frozen tissue recovery was between 1 – 5 hours. After recovery, samples were immediately placed into a chilled mixture of Dulbecco’s Modified Eagle Medium preservative and antibiotics and then shipped. Shipment time ranged from 24 – 36 hours before arrival and immediate testing.”*

21. “Line 354: Please clarify that it is ballistic gelatine that was used and what type of gelatine, of what concentration?”

    Response*:* As mentioned in the Methods section, a ballistic gel block was used in the reported experiments as backing behind the full-thickness tissue samples (lines 458-9). The gel block was comprised of pork gelatin, and while the manufacturer (BallisticsProGel; BCI) does not report concentration, we expect it is 10% gelatin based on observed stiffness while handling. Due to the low projectile velocity and mass, and use of full-thickness skin sample with subcutaneous fat, we do not

expect the gel stiffness to significantly affect the reported results. In the Methods Section, we have included clarification that pork gelatin from BallisticsProGel was used (line 459).

22. "Line 354: Was the skin sample mounted under tension? How was the natural skin tension accounted for? Please comment on the boundary condition of the skin sample in the experiment."

    ***Response**:* The skin was mounted under tension by using T-pins. Natural skin tension was approximated by stretching the skin just enough to remove large undulations or wrinkles and ensure a relatively flat skin surface. T-pins were spaced around the entire boundary of the tissue with a 2 – 3 cm interval to prevent large motion of the boundary. Impacts near the skin edge were evaluated and discarded if obvious edge effects were observed such as directionality of the wound normal to the edge. Due to the size of skin samples and low KED of projectile impacts, most impacts did not interact with the tissue boundary. This clarification has been added to the Methods section:

    At lines 459-64: *"Tension approximating natural skin tension was applied to the skin during mounting with T-pins to remove large tissue undulations and wrinkles and ensure a relatively flat test surface. T-pins were spaced around the tissue edge at an interval of 2 – 3cm to prevent large motion of the tissue boundary. Impacts near the skin edge were evaluated and discarded if obvious edge effects were observed such as directionality of the wound normal to the edge."*

23. "Line 356-365:
    What was the range of projectile impact velocity used in this study? What was the mass of the projectiles? Are they comparable to the study by Breeze et al. mentioned in the Discussion? Please comment on the relevance of the impact conditions used in this study to clinical cases and realistic scenarios."

    ***Response**:* The range of studied projectile velocity was 30 – 190 ms^{-1}. As already described in the Introduction (lines 117-9), these velocities are highly relevant since fragment velocities are expected to decrease rapidly during flight due to non-aerodynamic shapes. In the already referenced work by Champion et al. (line 119), Figure 3 shows the exponential decay of typical fragment velocities dependent on distance from the blast. A significant portion of fragment travel occurs at velocities of 200ms^{-1} and lower.

    The mass of projectiles was 0.34g for 4.4mm diameter steel spheres, 0.51g for 5.0mm diameter steel spheres, 0.12g for silica spheres, and 0.21g for Zn cylinders. These masses are highly comparable to the range of 0.49g or less mentioned in the report by Breeze et al. The mass values for projectiles have been added into the Methods section, followed by a brief comment on their comparability to reported fragment masses:

    At lines 476-7: *"Projectile masses are comparable to reported masses of the most common blast debris and fragments[53]."*

24. "Please clarify how the yaw was controlled and measured, and how it was taken into account for cross-section KE calculations."

***Response**:* Projectile yaw was not directly controlled due to limitations in the kinetic impact system, and was instead qualitatively evaluated through high-speed imaging of impacts. We believe this more closely resembles fragment impact in real blast events given the random nature of blast fragment shape, material, and initial acceleration, as discussed previously in lines 372-4. Additionally, yaw evaluation instead of control still enables the development of a high-fidelity FE model in which yaw may be easily controlled to probe specific impact parameters. How cylinder yaw was accounted for in cross-section KE calculations is discussed in response to Comment #8 from Reviewer #2.

Reviewer #3

1. “The authors state that "typical wound management involves antibiotics" (rows 62-63). This is not true. Antibiotics are generally given as prophylactic therapy to prevent infection in contaminated wounds, but not routinely in the case of relatively superficial (shallow) wounds, such as those under study. In reference #21 of the manuscript (Singer & Dagum, 2008), under "General Principles of Care", it is stated that "Prophylactic systemic antibiotics should not be used routinely". Also, in reference #22 (Trott, 2012), it is stated on page 3 "Currently, there are no solid, evidenced-based data showing that antibiotics prevent traumatic skin-wound infections", and also on page 225 "For small, uncomplicated, minor, non-bite wounds and lacerations, there is no convincing clinical evidence that systemic antibiotics provide protection against the development of wound infection".

   With respect to facial injuries, in "Maxillofacial Trauma and Esthetic Facial Reconstruction", 2nd edition (by Ward Booth, Elsevier; 2012), on page 432 it is stated that "Routine systemic antibiotics are not necessary. The use of antibiotics should be tailored based on host defense status, degree of contamination, mechanism of injury, and likelihood of wound infection.[...] Prophylactic antibiotics are necessary if the wound is expected to be contaminated, such as animal bites or wounds that communicate with the oral cavity".

   The use of antibiotics in skin wounds from debris and small projectiles depends on the depth of the wound (relatively superficial skin wounds such as abrasion injuries, although contaminated, they do not predispose to deep infection) and whether attempts to remove foreign bodies from facial wounds (in the case of traumatic tattooing) are undertaken carrying the risk of further contamination of a superficially contaminated wound.

   Regarding skin war wounds in general, In "Military Dermatology" (1994, United States Army, https://irp.fas.org/doddir/milmed/milderm.pdf), on page 149, it is stated "The use of antibiotics to prevent clinically apparent infections may be helpful in some instances.’ ”

   Response: We thank the reviewer for providing this clinical perspective on wound management and the proper use of antibiotics along with the references. We have modified the introductory paragraph and other mentions of infection accordingly:

   At lines 63-9: *“Skin injuries resulting from dynamic impact, particularly those involving extremities, often present severe bleeding and possible infection risks… Typical wound management can be costly and requires intensive wound debridement protocols to remove colonized necrotic tissue and foreign bodies.”*

At lines 119-21: *"Some of these projectiles or accompanying debris can be biologically active, causing increased contamination of distributed partial-thickness skin wounds*[54,55]*."*

At lines 304-6: *"Considering low-angle impacts also cause large injury areas over all energies, these partial avulsions present a substantial threat of contamination and bleeding to the exposed dermal tissue*[18,22,63,64]*."*

2. "The interesting observations regarding high-angle spherical projectile impact that it is associated with creation of a large temporary crater on the skin surface, while the permanent wound is smaller in diameter than the projectile itself (because of the skin elasticity), also associated with small lacerations in the epidermis (apparently from the stretching effect during the impact) are in accordance with similar effects produced at the site of entrance of bullets. Please refer to Thali et al., "A study of the morphology of gunshot entrance wounds, in connection with their dynamic creation, utilizing the ''skin–skull–brain model''", Forensic Science International (2002) 125:190–194 (https://pubmed.ncbi.nlm.nih.gov/11909662/). So I would recommend that the authors comment on these similarities, which are both consistent with the skin properties and the process of energy transfer by the projectile upon impact."

   Response*:* We thank the reviewer for the valuable reference, and for the useful comparison between our observations of epidermal lacerations and the findings reported by Thali et al. concerning the "abrasion ring" or "dilation mark" in gunshot wounds. We agree the temporary impact crater causes such large elastic tissue deformation and stretch that permanent epidermal damage results. We have including the following comments to the Discussion to highlight this comparison along with the role of skin elasticity in energy dissipation and the size of permanent damage:

   At lines 313-23: *"Interestingly, the epidermal lacerations observed after high-angle impact may correspond to similar skin damage described as the "abrasion ring" or "dilation mark" in the case of perforating gunshot wounds*[66]*. In this case, epidermal damage occurs due to the substantial amount of elastic tissue stretch that arises during temporary impact crater formation (Fig. 2f). The temporary wound crater or cavity is a known impact energy dissipation mechanism of the skin and soft tissue that may also cause significant internal damage, especially during perforating impacts*[29–31]*. The elastic and viscoelastic properties of skin are known to underlie temporary crater formation, and also explain why permanent damage from high-angle impacts is of smaller diameter than the projectile itself in observed partial-thickness and perforating cutaneous damage (Fig.4f)*[30,66]*."*

3. "The authors use the term cross-section kinetic energy (KE) measured in joules per square centimeter (kinetic energy per unit area). A more common term for this measure used in the wound ballistics literature, which is an expression of the penetration capacity of the projectile, is the kinetic energy density. Please refer to Kneubuehl's "Wound Ballistics" (Springer, 2011), and also to the chapter "Personal Armour Used by UK Armed Forces and UK Police Forces" by Lewis et al., in "Ballistic Trauma", 4th edition (Breeze et al., Springer, 2017), and also to Caister et al., "The ballistic performance of bone when impacted by fragments", International Journal of Legal Medicine (2020) 134:1387–1393 (https://pubmed.ncbi.nlm.nih.gov/32358725/)."

***Response**:* We thank the author for the references reinforcing this point concerning terminology. We agree that changing the terminology to “kinetic energy density” or “KED” would better reflect literature terminology. We have changed “cross-section kinetic energy” to “kinetic energy density” in the manuscript and included the description of KED as the “projectile kinetic energy per cross-section unit area” at first use of the term as is typically cited in the literature.

4. “The book "Wounds and Lacerations: Emergency Care and Closure" by Alexander Trott, which appears as reference #22, is in its 4th edition.”

   ***Response**:* We have updated the reference to reflect the book is in its 4th edition.

REVIEWERS' COMMENTS:

Reviewer #1 (Remarks to the Author):

I thank the authors for their responses on my comments and questions about the manuscript submitted. And, the authors have answered almost all the questions, but for the last question, the response is not complete.
The material model is explained but regarding the values used for the parameters, it is said to search in in the bibliography, and there are many different values.
I think it would be better if the authors include a table with all the values used in their simulations to be able to reproduce their work.
Also, about the simulation, it would be a goog idea to mention the element number or type and the boundary conditions used, and even if they use some specific values for the hourglass parameters or shear locking effect in explicit analysis.

Reviewer #2 (Remarks to the Author):

Dear Authors,

Thank you very much for responding to my comments and for the revised manuscript.
My concerns have been addressed through the additional details and discussions.

I would like to endorse the publication of this manuscript in its current form.

With best regards,

Thuy-Tien N Nguyen

Reviewer #3 (Remarks to the Author):

The authors have responded satisfactorily and exhaustively to all the comments made by the reviewers, with the comments of the other two reviewers being both important and extremely enlightening, and thus the revision made has greatly enhanced the power of the manuscript. Therefore, I would recommend acceptance for publication by the journal.

We thank the reviewers for their comments on our manuscript submitted to Communications Engineering.

The reviewer's comments are listed below together with our responses.

Modifications within the manuscript submitted separately are highlighted in red.

Reviewer #1

1. "The material model is explained but regarding the values used for the parameters, it is said to search in in the bibliography, and there are many different values. I think it would be better if the authors include a table with all the values used in their simulations to be able to reproduce their work."

   ***Response**:* Parameter values used in the Ogden hyperelastic model and in the Prony time series expansion have been included in the new Table 1 and Table 2, respectively. In the case of the Gasser-Ogden-Holzapfel model used for the dermis the material parameters were included in the Methods section:

   At lines 507-8: *"In this work C was set to 0.01MPa, k_1 was set to 2.2MPa, k_2 was set to 0.86, and κ was set to 0.27."*

Table 1. Ogden hyperelastic model coefficients

Skin Layer	μ (MPa)	α
Epidermis	2.92	6.35
Hypodermis	0.0104	13.57

Table 2. Prony time series expansion coefficients

Skin Layer	g_1	τ_1	g_2	τ_2	g_3	τ_3
Dermis	0.09	1.44	0.15	8.63	0.17	52
Hypodermis	0.09	0.294	0.215	9.557	-	-

2. "Also, about the simulation, it would be a goog idea to mention the element number or type and the boundary conditions used, and even if they use some specific values for the hourglass parameters or shear locking effect in explicit analysis."

   ***Response**:* Additional details regarding simulation elements, mesh, boundary conditions, and explicit analysis have been added to the Methods section:

At lines 517-26: *"Displacement boundary conditions were implemented along the base of the hypodermis in the z-plane and perpendicularly along the side surfaces in the x-direction and y-direction. 8-node linear hex elements with reduced integration (C3D8R) and enhanced hourglass control were used to mesh the model, and element size was reduced until mesh convergence was achieved. Mesh density was largest at the area of impact, and the smallest element size was 0.02mm for the SC, 0.05mm for epidermis and dermis, and 0.1mm for the hypodermis. A dynamic explicit analysis was performed for a 1.5ms duration, calculation of the minimum time step was automated to ensure numerical convergence, and dynamic mass scaling was implemented to reduce computational cost, ensuring that energy conservation was maintained in the process."*

We appreciate your comments on our work.

Sincerely,
Reinhold H. Dauskardt
Professor
